# Tetracyclines improve experimental lymphatic filariasis pathology by disrupting interleukin-4 receptor–mediated lymphangiogenesis

**DOI:** 10.1172/JCI140853

**Published:** 2021-03-01

**Authors:** Julio Furlong-Silva, Stephen D. Cross, Amy E. Marriott, Nicolas Pionnier, John Archer, Andrew Steven, Stefan Schulte Merker, Matthias Mack, Young-Kwon Hong, Mark J. Taylor, Joseph D. Turner

**Affiliations:** 1 Centre for Drugs & Diagnostics, Department of Tropical Disease Biology, Liverpool School of Tropical Medicine, Liverpool, United Kingdom.; 2 Institute for Cardiovascular Organogenesis and Regeneration, Faculty of Medicine, Westfälische Wilhelms-Universität Münster, Münster, Germany.; 3 Universitätsklinikum Regensburg, Regensburg, Germany.; 4 Department of Surgery, Norris Comprehensive Cancer Center, Keck School of Medicine, University of Southern California, Los Angeles, California, USA.

**Keywords:** Infectious disease, Inflammation, Lymph, Parasitology, Th2 response

## Abstract

Lymphatic filariasis is the major global cause of nonhereditary lymphedema. We demonstrate that the filarial nematode *Brugia malayi* induced lymphatic remodeling and impaired lymphatic drainage following parasitism of limb lymphatics in a mouse model. Lymphatic insufficiency was associated with elevated circulating lymphangiogenic mediators, including vascular endothelial growth factor C. Lymphatic insufficiency was dependent on type 2 adaptive immunity, the interleukin-4 receptor, and recruitment of C-C chemokine receptor-2–positive monocytes and alternatively activated macrophages with a prolymphangiogenic phenotype. Oral treatments with second-generation tetracyclines improved lymphatic function, while other classes of antibiotic had no significant effect. Second-generation tetracyclines directly targeted lymphatic endothelial cell proliferation and modified type 2 prolymphangiogenic macrophage development. Doxycycline treatment impeded monocyte recruitment, inhibited polarization of alternatively activated macrophages, and suppressed T cell adaptive immune responses following infection. Our results determine a mechanism of action for the antimorbidity effects of doxycycline in filariasis and support clinical evaluation of second-generation tetracyclines as affordable, safe therapeutics for lymphedemas of chronic inflammatory origin.

## Graphical abstract

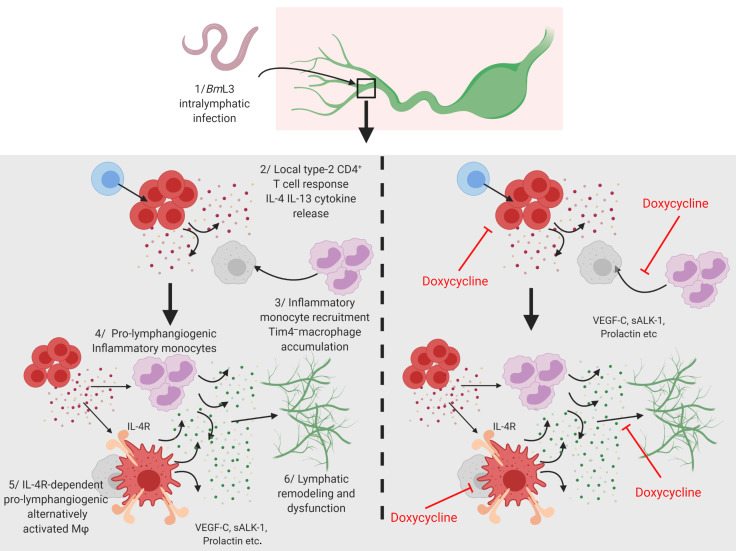

## Introduction

Lymphedema (LE) affects 200 million individuals worldwide (1). LE is caused by disruption of normal lymphatic function whereby return drainage of fluid, proteins, fats, and immune cells (lymph) is impaired (2). LE is either hereditary, caused by mutations in genes controlling lymphatic development, or nonhereditary, caused by infection, trauma, or surgical removal of lymphatics to prevent cancer metastasis (2, 3). The major cause of secondary LE is lymphatic filariasis (LF), a neglected tropical disease affecting an estimated 67 million people, with a further 890 million at risk (4). Filarial LE causes life-long physical and associative mental disability (5), ranking LF as the fourth highest contributor to global disability-adjusted life-years. Tangible progress has been made in LF elimination via mass drug administration of antifilarial drugs, effectively halving the number of active infections between 2000 and 2013 (4), whereas the number of LE patients remained static at 40 million over the same time period. Current treatment for filarial LE is limited to morbidity management and disability prevention, which involves an array of hygienic measures and implementation of physiotherapy in the household (6). No chemotherapeutic interventions are indicated for filarial LE. However, antibiotics are recommended to treat secondary skin bacterial infections, which can reduce the frequency of periodic inflammatory episodes known as acute dermatolymphangioadenitis (ADLA), a form of cellulitis. In a recent placebo-controlled clinical trial, while both amoxicillin (the standard antibiotic treatment for ADLA) and doxycycline reduced the frequency of ADLA, doxycycline also showed surprising efficacy in reversing LE grade (7).

Lymphatic remodeling is a hallmark of filarial LE, with clearly established evidence from in vitro (8, 9)*,* in vivo (10–13), and clinical (14–16) studies. How lymphatic remodeling develops and its role in LF pathology is poorly understood.

In this study, we developed a murine hind-limb model of filarial infection, utilizing longitudinal intravital imaging to demonstrate that filarial infective larvae induce rapid lymphatic alterations associated with induction of lymphatic insufficiency. We demonstrate that early filarial lymphatic pathology is primarily host-immune driven, characterizing an interleukin-4 receptor (IL-4R) type 2–dependent axis involving recruitment of inflammatory monocytes and alternatively activated macrophages (AAMΦs) that promote the development of lymphatic disease. We demonstrate that second-generation tetracyclines can target multiple aspects of this pathway to ameliorate lymphatic pathology.

## Results

### Brugia malayi infection induces lymphatic remodeling and dysfunction.

**Figure 1:**
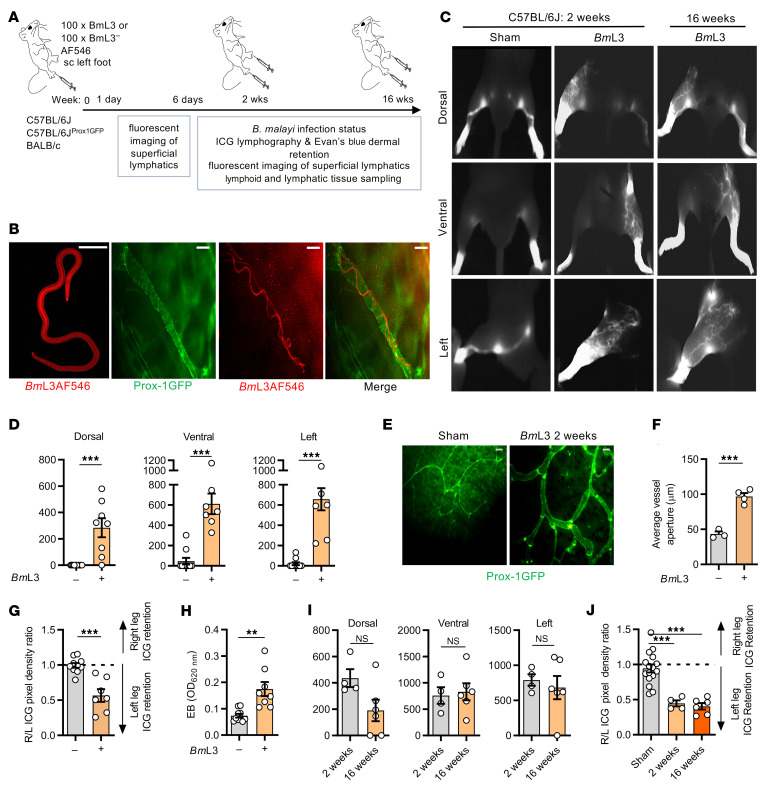
Filarial lymphatic infection induces persistent lymphatic pathology. (**A**) Schematic of hind-limb filarial infection model. (**B**) Representative images of in vitro (left panel) or intralymphatic Alexa Fluor 546–labeled (AF546) *Bm*L3 larvae in C57BL/6J Prox-1^GFP^ mice, 1 dpi. Scale bars: 20 μm. (**C**) Representative PDE intravital images of sham-infected and *Bm*L3-infected C57BL/6J mice, 14 dpi. (**D**) Quantified aberrant lymphatics from PDE imaging (*n* = 10 sham, *n* = 8 *Bm*L3). (**E**) Representative epifluorescence micrographs of dermal lymphatics and (**F**) average dermal lymphatic vessel aperture in Prox-1^GFP^ mice 14 dpi (*n* = 3 Sham, *n* = 4 *Bm*L3). Scale bars: 200 μm. (**G**) Quantified hind-limb ICG dye retention from PDE imaging expressed as a ratio of fluorescence in the right (R, uninfected) vs. the left (L, infected) hind limb (*n* = 10 sham, *n* = 8 *Bm*L3). (**H**) Evan’s blue left-hind-limb dermal retention (*n =* 9 sham, *n =* 8 *Bm*L3). (**I**) Aberrant lymphatics and (**J**) hind-limb ICG retention comparing 2- and 16-week-old infections (*n =* 15 sham, *n =* 4 *Bm*L3 at 2 weeks after infection, *n =* 4 at 16 weeks after infection). Histograms show the mean ± SEM. Data were pooled from 2–3 individual experiments (**D** and **G**–**J**) or 1 experiment (**F**). **P < 0.01, ***P < 0.001 by 2-tailed Student’s *t* test (**D** and **F**–**I**) or 1-way ANOVA with Tukey’s multiple-comparison post hoc test (**J**). NS, not significant.

We developed a murine lymphatic pathology model whereby C57BL/6J mice were administered with subcutaneous inoculations of *Brugia* *malayi* infective third-stage larvae (*Bm*L3) to the left hind limb (Figure 1A). We confirmed that filarial larvae established intralymphatic infections by imaging motile, fluorescently labeled *Bm*L3 within GFP-tagged Prox-1^+^ collecting vessels (lymphangions) of the infected hind limb (Figure 1B and Supplemental Video 1; supplemental material available online with this article; https://doi.org/10.1172/JCI140853DS1). Motile *Bm*L3 could be observed within superficial dermal lymphatics from 3 hours to 4 days after infection. Near-infrared (NIR) intravital indocyanine green (ICG) lymphography was undertaken to investigate the impact of *B*. *malayi* larval infection on lymphatic structure and function (Figure 1A, Supplemental Figure 1, and Supplemental File 1). Clinical ICG lymphography has characteristic “splash,” “stardust,” and “diffuse” dermal backflow patterns, and visualization of tortuous collateral lymphatics, associated with onset of LE in patients (17). At 2 weeks after *B*. *malayi* infection, we observed the presence of all 3 dermal backflow patterns and tortuous collateral lymphatic development (Figure 1C and Supplemental Figure 2, A and B). By image analysis we determined that *Bm*L3-infected C57BL/6J mice displayed significant levels of lymphatic remodeling in dorsal, lateral, and ventral aspects of the infected limb (Figure 1, C and D). Remodeling was pronounced at sites proximal to initial invasion of the superficial lymphatics, although by this time point there was no evidence of motile intralymphatic larvae. By epifluorescence imaging, we could detect significant, mean 2-fold dilations of Prox-1^+^ lymphatic vessels at 2 weeks after infection (Figure 1, E and F). By comparing ICG dermal backflow in infected and uninfected limbs, significant ICG retention was evident in the infected limbs, compared with sham controls (Figure 1G). Further, in an Evan’s blue (EB) dermal retention assay (Supplemental Figure 1), significant EB accumulation in the skin of *Bm*L3-infected limbs was discerned (Figure 1H). Repeat experiments using BALB/c mice demonstrated that all aspects of lymphatic pathology were reproducible in this background strain, although to a generally lower degree of severity (Supplemental Figure 2).

Because inbred mice mount an effective adaptive immune response to control *B*. *malayi* infection before chronic adult intralymphatic filarial parasitism can become established (18), we next investigated if infection-induced lymphatic remodeling and dysfunction resolved after clearance of filarial infection. *Bm*L3-infected mice imaged at 16 weeks after infection retained backflow and tortuous lymphatic patterning, with no significant decline in lymphatic remodeling or levels of lymphatic insufficiency, compared with 2 weeks after infection (Figure 1, C, I, and J). At 16 weeks after infection, there was no evidence of active intralymphatic adult parasitism or circulating microfilariae, indicating that lymphatic pathology persists long after initial induction by filarial infection.

**Figure 2:**
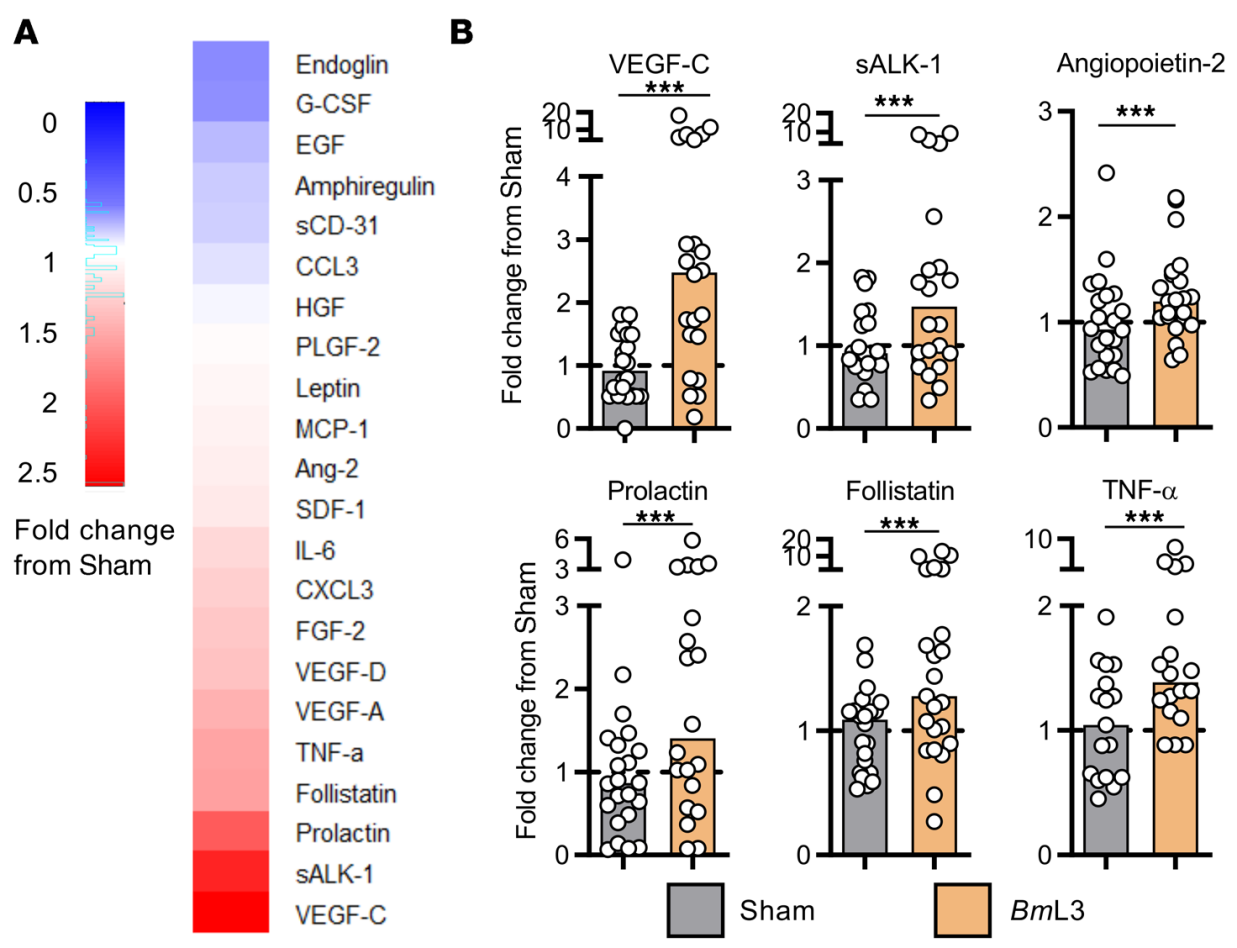
Filarial infection induces increases in circulating lymphangiogenic molecules. (**A**) Circulating levels of lymphangiogenic molecules. Heatmap plots median fold-change in analyte from sham-infected mouse group; red = fold-increase from sham-infected, blue = fold-decrease (*n =* 21 sham; *n =* 22 *Bm*L3). (**B**) Circulating lymphangiogenic molecule concentrations from **A** for analytes achieving statistical significance. Histograms show the medians. Data were pooled from 4 individual experiments. ***P < 0.001 by Mann-Whitney test.

To explore host molecular mechanisms mediating filarial lymphatic pathology, we compared circulating plasma concentrations of a focused array of angiogenic/lymphangiogenic factors between *Bm*L3- and sham-infected cohorts at 14 days postinfection (dpi). A milieu of lymphangiogenic factors were upregulated in *Bm*L3-infected mice, including vascular endothelial growth factor C (VEGF-C), soluble activin receptor–like kinase 1 (sALK-1), and prolactin (Figure 2, A and B). As VEGF-C is a well-characterized primary lymphangiogenic mediator (19), we investigated the impact of isolated VEGF-C delivery to the hind-limb skin-draining lymph nodes (sdLNs).

We administered a VEGF-C–expressing adenoviral vector (adVEGF-C) to increase local VEGF-C signaling in the same anatomical areas exposed to *Bm*L3 infection. adVEGF-C–treated groups displayed significantly higher levels of both lymphatic remodeling and insufficiency, compared with both naive mice and control mice treated with GFP-expressing adenoviral vector (adGFP), with mid-dose adVEGF-C administration recapitulating magnitudes of lymphatic remodeling and pathology comparable to 14-dpi *Bm*L3-infected mice (Supplemental Figure 3).

### Filarial lymphatic pathology is dependent on IL-4R type 2 adaptive immune responses.

**Figure 3:**
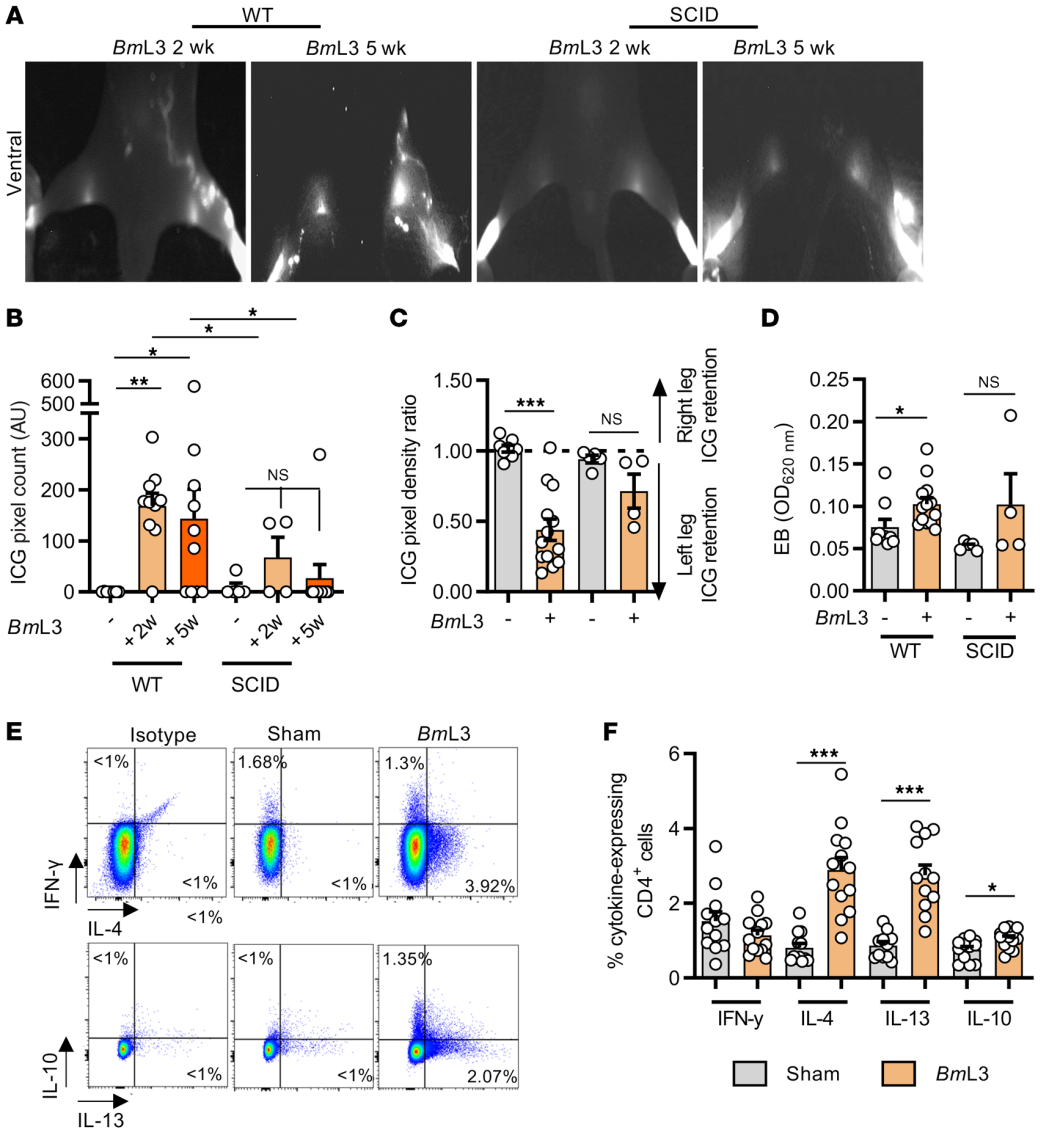
Filaria-associated lymphatic pathology is dependent on type 2 adaptive immunity. (**A**) Representative PDE intravital images and (**B**) aberrant lymphatic quantification of WT BALB/c and SCID mice at 2 and 5 weeks postinfection (wpi) (*n =* 8 WT sham, *n =* 10 WT *Bm*L3 at 2 wpi and 5 wpi, *n =* 5 SCID sham, *n =* 4 SCID *Bm*L3 at 2 wpi, *n =* 10 SCID *Bm*L3 at 5 wpi). (**C**) Hind-limb ICG dye retention and (**D**) Evan’s blue left-hind-limb dermal retention in WT and SCID mice, 14 dpi (*n =* 7 WT sham, *n =* 14 WT *Bm*L3, *n =* 5 SCID sham, *n =* 4 SCID *Bm*L3). (**E**) Representative flow cytometry plots and (**F**) quantified cytokine production within sdLN CD4^+^ T cells from C57BL/6J mice, 14 dpi (*n =* 12 sham, *n =* 13 *Bm*L3). Data are cytokine-expressing cells as a proportion of total CD4^+^ T cells. Histograms show the mean ± SEM. Data were pooled from 2 individual experiments (**B** and **F**) or a single experiment (**D**). *P < 0.05, **P < 0.01, ***P < 0.001 by 1-way ANOVA with Tukey’s multiple-comparison post hoc test between marked groups. NS, not significant.

**Figure 4:**
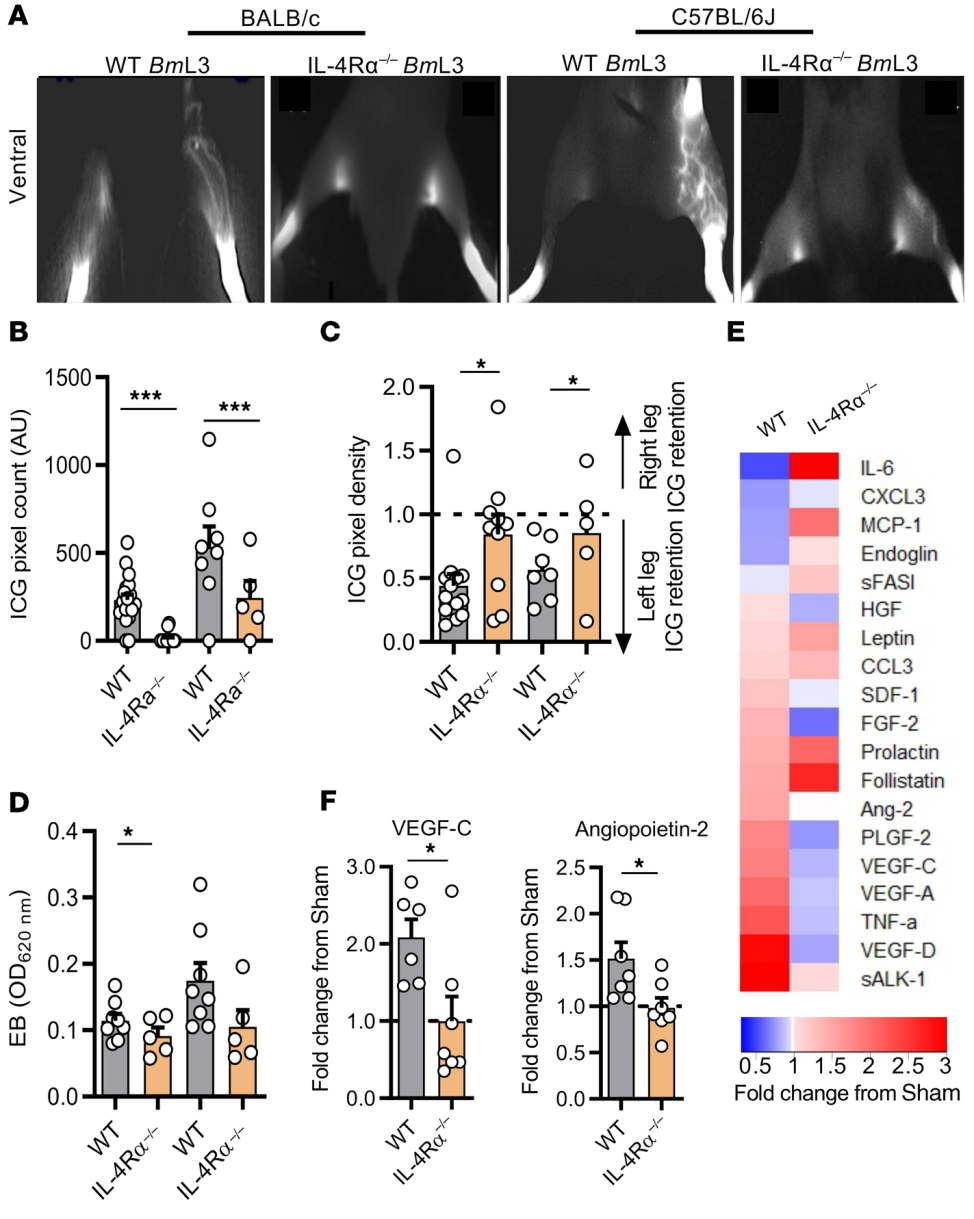
Filaria-associated lymphatic pathology is dependent on IL-4 receptor immune responses. (**A**) Representative images, (**B**) quantified aberrant lymphatics, and (**C**) quantified hind-limb ICG dye retention in WT and IL-4Rα^–/–^ BALB/c and C57BL/6J mice, 14 dpi (*n =* 20 WT BALB/c sham, *n =* 21 WT BALB/c *Bm*L3, *n =* 8 IL-4Rα^–/–^ BALB/c sham, *n =* 15 IL-4Rα^–/–^ BALB/c *Bm*L3, *n =* 10 WT C57BL/6J sham, *n =* 10 WT C57BL/6J *Bm*L3, *n =* 5 IL-4Rα^–/–^ sham and IL-4Rα^–/–^ *Bm*L3). (**D**) Evan’s blue left-hind-limb dermal retention in WT and IL-4Rα^–/–^mice, 14 dpi (*n =* 8 WT BALB/c *Bm*L3, *n =* 5 IL-4Rα^–/–^ BALB/c *Bm*L3, *n =* 8 WT C57BL/6J *Bm*L3, *n =* 5 IL-4Rα^–/–^ sham and IL-4Rα^–/–^ *Bm*L3). (**E**) Comparison of circulating levels of lymphangiogenic molecules between WT C57BL/6J WT and IL-4Rα^–/–^ *Bm*L3-infected mice, 14 dpi. Heatmap plots median fold-change in analyte from sham-infected mouse group; red = fold-increase from sham-infected, blue = fold-decrease (*n =* 6 WT *Bm*L3, *n =* 7 IL-4Rα^–/–^ *Bm*L3). (**F**) Plots of lymphangiogenic analytes achieving statistical significance. Histograms show the mean ± SEM. Data were pooled from 2–3 individual experiments. *P < 0.05, ***P < 0.001 by 1-way ANOVA with Tukey’s multiple-comparison post hoc test (**B**–**D**) or 2-tailed Student’s *t* test between marked groups (**F**).

Previous clinical studies have demonstrated a link between symptomatic LF and enhanced parasite-specific host adaptive immune responses (20, 21). In mice, a polarized type 2 adaptive immune response coordinates effective eosinophil-mediated immunity against larval stage filariae (22). We investigated the role of adaptive immunity by comparing magnitudes of lymphatic remodeling and insufficiency between WT and severe combined immunodeficient (SCID) mice lacking functional B and T lymphocytes. *Bm*L3-infected CB.17 (BALB/c congenic) SCID mice displayed muted levels of lymphatic remodeling that were not significantly different compared with sham controls and significantly lower than corresponding WT BALB/c infections assessed at either 2 or 5 weeks after infection (Figure 3, A and B). Concomitantly, no significant difference in lymphatic insufficiency was observed between sham and infected SCID mice, judged by either ICG or EB dermal backflow at 2 weeks after infection (Figure 3, C and D). We then characterized the localized CD4^+^ T cell adaptive immune response in sdLNs and major afferent lymphatic collecting vessels proximal to filaria-parasitized and -remodeled lymphatic tissues, utilizing intracellular cytokine flow cytometry (Supplemental Figure 4). Significant expansions of type 2 IL-4– and IL-13–secreting CD4^+^ T cells were observed in sdLN single-cell suspensions derived from *Bm*L3-infected mice at 14 dpi and CD4^+^ secretion levels of the regulatory-type cytokine IL-10 was also increased, while local secretion levels of the type 1 cytokine IFN-γ remained unaltered following infection (Figure 3, E and F). Subsequently, we tested whether ablation of type 2 immune signaling would affect the severity of filarial lymphatic pathology, utilizing IL-4Rα–deficient mice, which are unable to respond to either IL-4 or IL-13. Following *Bm*L3 infection, IL-4Rα–knockout (IL-4Rα^–/–^) mice, on either the BALB/c or C57BL/6J background, exhibited significantly diminished lymphatic remodeling and lymphatic dysfunction (Figure 4, A–D). Levels of circulating lymphangiogenic mediators were also significantly abrogated in IL-4Rα^–/–^ *Bm*L3–infected mice, notably VEGF-C and angiopoietin 2 (Ang-2) (Figure 4, E and F). These data indicate a functional role for IL-4R–dependent type 2 adaptive immune responses in the induction of early filarial lymphatic pathology.

### Prolymphangiogenic inflammatory monocytes and AAMΦs are mediators of filarial lymphatic dysfunction.

**Figure 5:**
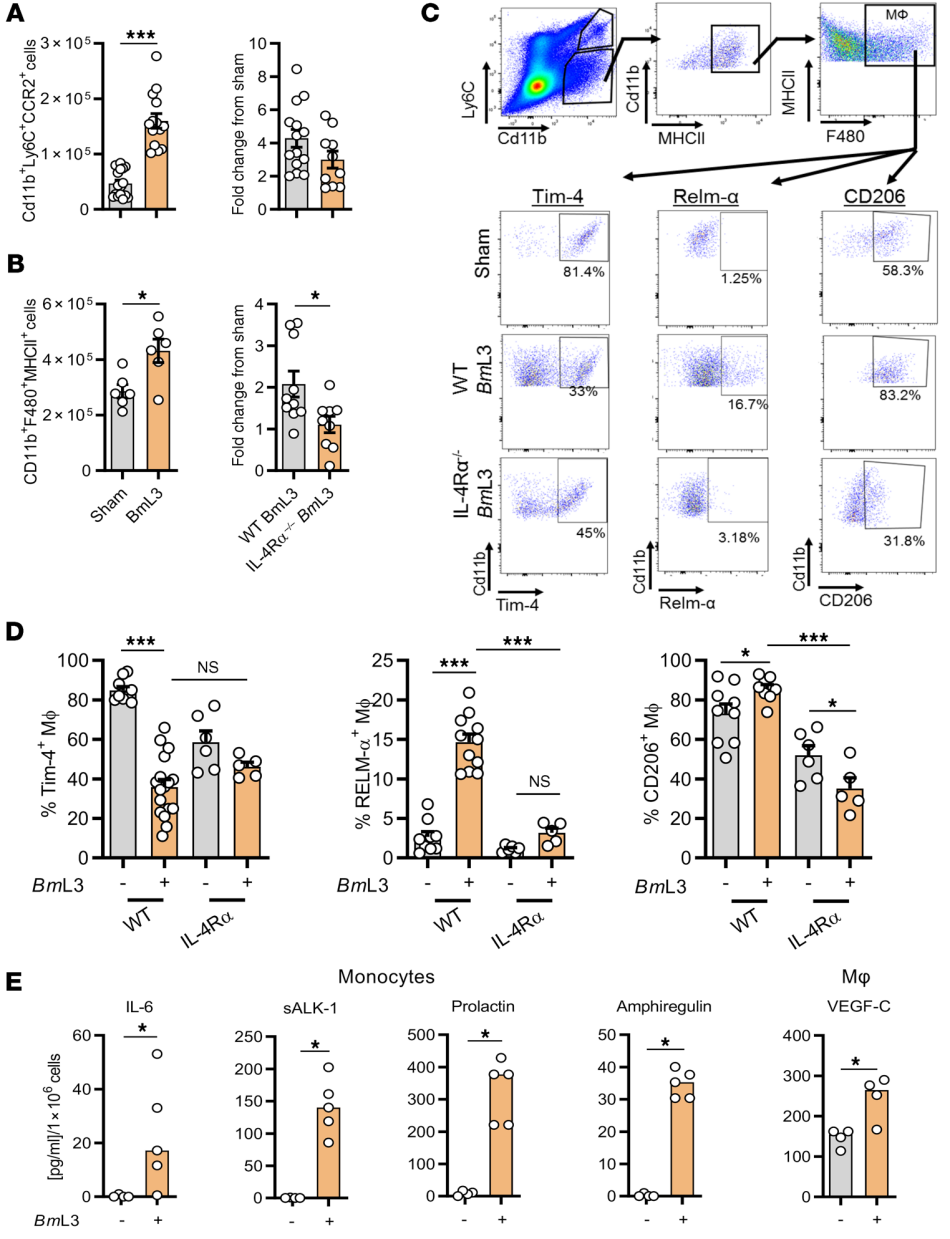
*Bm*L3 infection drives lymphatic monocyte recruitment and expansion of alternatively activated, prolymphangiogenic macrophages. (**A**) Numbers of CD11b^+^Ly6C^+^CCR2^+^ inflammatory monocytes (*n =* 16 sham, *n =* 14 WT *Bm*L3, *n =* 10 IL-4Rα^–/–^ *Bm*L3) or (**B**) Cd11b^+^F4/80^+^MHCII^+^ MΦs (*n =* 6 sham, *n =* 10 WT *Bm*L3; *n =* 9 IL-4Rα^–/–^ *Bm*L3) derived from sdLNs and major lymphatic channels in C57BL/6J mice, 14 dpi. Data are total cell numbers or fold-change from relevant sham controls. (**C**) Representative flow plots of lymphatic MФ phenotyping in sham- and *Bm*L3-infected mice. Percentages are proportions of total CD11b^+^F4/80^+^MHCII^+^ MΦs. (**D**) CD206^+^, RELM-α^+^, and Tim-4^+^ MΦ expression in WT and IL-4Rα^–/–^ sham- and *Bm*L3-infected mice (*n =* 9 WT sham, *n =* 9–17 WT *Bm*L3, *n =* 6 IL-4Rα^–/–^ sham, *n =* 5 IL-4Rα^–/–^ *Bm*L3). (**E**) Significant changes in specific lymphangiogenic molecules secreted following 72-hour ex vivo incubation of FACS-isolated lymphatic monocytes or MΦs derived from sham- or *Bm*L3-infected mice. Secretion is normalized to analyte concentration/1 × 10^6^ cells (*n =* 4 sham, *n =* 5 *Bm*L3). Data were pooled from 2–3 individual experiments. Histograms show the mean ± SEM (**A**–**D**) or median (**E**). *P < 0.05, ***P < 0.001 by 2-tailed Student’s *t* test (**A** and **B**), 1-way ANOVA with Tukey’s multiple-comparison post hoc test (**D**), or Mann-Whitney test (**E**). NS, not significant.

**Figure 6:**
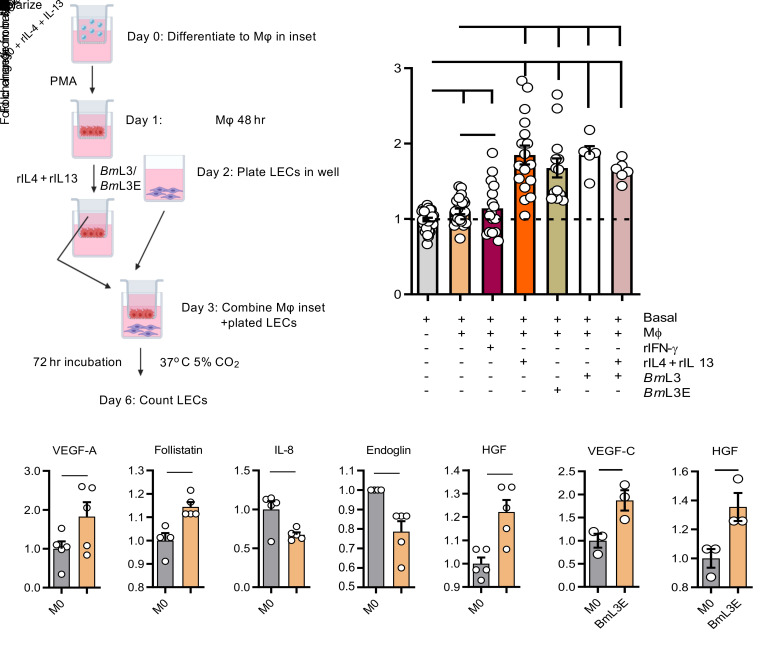
Human monocyte–derived MΦs conditioned with rIL-4 +rIL-13 and/or filarial *Bm*L3 results in a lymphangiogenic phenotype that induces proliferation of lymphatic endothelium. (**A**) Schematic of in vitro human dermal lymphatic endothelial cells (LECs) cocultured with preconditioned human monocyte–derived MΦs. LEC proliferation was quantified 72 hours after addition of MΦ cocultures. (**B**) LEC proliferation following monocyte-derived-MΦ cocultures conditioned with recombinant IL-4 and IL-13 (rIL-4+rIL-13), live *Bm*L3 (*Bm*L3), *Bm*L3 larval extract (*Bm*L3E), rIL+4+rIL-13+*Bm*L3, or unconditioned MΦs, expressed as fold-change from mean basal LEC enumerations. (**C**) Concentrations of lymphangiogenic molecules 72 hours after MΦ culture in the absence or presence of rIL-4+rIL-13 or (**D**) *Bm*L3E (MΦ+*Bm*L3E), expressed as fold-change from mean unstimulated MΦ levels (M0 = unstimulated THP-1–differentiated MΦs). Histograms show the mean ± SEM. Data were pooled from 3 individual experiments (**A** and **B**) or a single experiment (**C** and **D**). *P < 0.05, ***P < 0.001 by 1-way ANOVA with Tukey’s multiple-comparison post hoc test (**B**) or 2-tailed Student’s *t* test between indicated groups (**C** and **D**). NS, not significant.

We investigated the contribution of local cellular inflammatory responses in mediating filarial lymphatic pathology. By immunophenotyping the sdLNs and major afferent lymphatic collecting vessels proximal to *Bm*L3 inoculation sites, we determined significant expansions of CD11b^+^Ly6C^+^CCR2^+^ inflammatory monocyte and CD11b^+^F4/80^+^MHCII^+^ MΦ populations (Figure 5, A and B), significant eosinophilic and neutrophilic granulocyte recruitment, and T and B lymphocyte proliferation (Supplemental Figure 5). In the absence of functional IL-4Rα signaling, a slight decrease in monocyte recruitment was observed and lymphatic tissue MΦ expansions were significantly impeded following filarial infection (Figure 5, A and B). A significant, 2-fold reduction in MΦs expressing the tissue residency marker Tim-4 (23), in filaria-infected WT but not IL-4Rα^–/–^mice, was apparent (Figure 5, C and D), suggestive of IL-4R–dependent recruitment of monocyte-derived MΦs within the expanded lymphatic tissue MΦ pool, after *Bm*L3 infection. Filarial infection–expanded lymphatic tissue MΦs also displayed significantly increased expression of the AAMФ markers RELM-α and CD206 (mannose receptor, a specific marker of alternative activation within monocyte-derived MΦs in cardiac and hepatic tissues; refs. 24, 25, and Figure 3, C and D). AAMΦ development after filarial infection in proximal lymphatic tissues was completely abrogated in the absence of intact IL-4R signaling (Figure 3, C and D). AAMΦ polarization is a well-characterized hallmark of filarial infection (26), and IL-4–stimulated AAMΦs are important in mediating filarial expulsion by sustaining recruitment of eosinophils (22). Because MΦs are potent cellular mediators of angiogenesis and lymphangiogenesis (27), we explored the lymphangiogenic phenotype of purified monocytes and macrophages from lymphatic tissues after filarial infection. FACS-isolated CD11b^+^Ly6C^+^CCR2^+^ inflammatory monocytes secreted significantly higher concentrations of prolactin, sALK-1, IL-6, and amphiregulin, while CD11b^+^F4/80^+^MHCII^+^ MΦs secreted significantly higher levels of VEGF-C compared with sham-infected controls (Figure 5E). In a tandem approach, we examined the direct prolymphangiogenic potential of type 2 cytokine– or filaria-stimulated human THP-1 monocyte–derived MΦs. For this purpose, we developed a human dermal lymphatic endothelial cell (LEC) proliferation assay following coculture with monocyte-derived MΦs preconditioned with recombinant (r) IFN-γ, rIL-4+rIL-13, and live *Bm*L3 or *Bm*L3 extract (*Bm*L3E) (Figure 6A). rIL-4+rIL-13–, live *Bm*L3–, and *Bm*L3E-conditioned MΦs mediated significant LEC proliferation compared with LEC cells cultured in basal media alone or in the presence of naive THP-1 monocyte–derived MΦs (Figure 6B). Analysis of conditioned media from rIL-4+rIL-13–stimulated monocyte-derived MΦs revealed significantly elevated levels of prolymphangiogenic mediators VEGF-A, follistatin, and human growth factor (HGF) (Figure 6C), while significantly elevated VEGF-C and HGF were observed in *Bm*L3E-pulsed MΦ–conditioned media (Figure 6D). This human cell coculture system confirmed that monocyte-derived MΦs exposed to both a type 2–dominated (IL-4+IL-13) microenvironment and filaria-specific antigenic stimuli polarize toward a lymphangiogenic phenotype, capable of inducing the proliferation of LECs.

**Figure 7:**
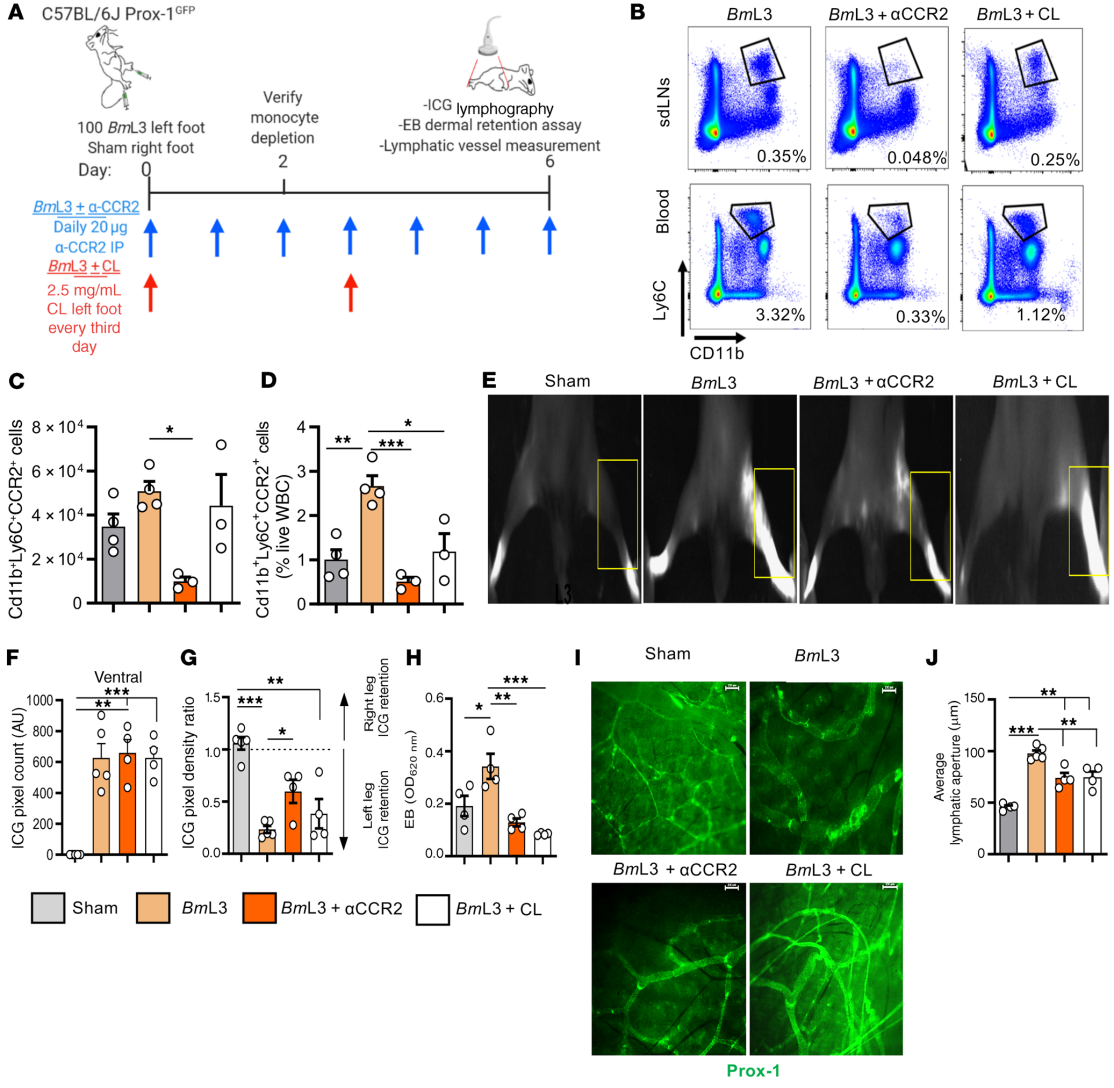
Depletion of CCR2^+^ monocytes or phagocytes significantly ameliorates filaria-induced lymphatic insufficiency. (**A**) Schematic of CCR2^+^ monocyte and phagocyte depletion regimens in *Bm*L3-infected C57BL/6J Prox-1^GFP^ mice. (**B**) Representative flow cytometry plots from *Bm*L3-infected mice or *Bm*L3-infected mice treated with either anti-CCR2 ablating antibody (*Bm*L3+αCCR2) or clodronate liposomes (*Bm*L3+CL), 2 dpi. Percentages are CD11b^+^Ly6C^+^ cells as a proportion of live cells. (**C**) CD11b^+^Ly6C^+^CCR2^+^ inflammatory monocytes isolated from hind-limb lymphatic tissues or (**D**) blood, derived from sham, *Bm*L3, *Bm*L3+αCCR2, or *Bm*L3+CL mice, 2 dpi. Data in **D** are reported as proportions of total white blood cells (WBC) (*n =* 4 sham and *Bm*L3, *n =* 3 *Bm*L3+αCCR2 and *Bm*L3+CL). (**E**) Representative PDE images of sham, *Bm*L3, *Bm*L3+αCCR2, and *Bm*L3+CL mice, 6 dpi. Yellow boxes highlight ICG retention (**F**) aberrant lymphatics, (**G**) hind-limb ICG retention, and (**H**) Evan’s blue dermal retention in sham, *Bm*L3, *Bm*L3+αCCR2, and *Bm*L3+CL mice, 6 dpi (*n =* 5 sham and *Bm*L3, *n =* 4 *Bm*L3+αCCR2 and *Bm*L3+CL). (**I**) Representative epifluorescence images of lymphatic vessels and (**J**) average lymphatic vessel aperture in sham, *Bm*L3, *Bm*L3+αCCR2, and *Bm*L3+CL mice, 6 dpi (*n =* 5 sham and *Bm*L3, *n =* 4 *Bm*L3+αCCR2 and *Bm*L3+CL). Scale bars: 200 μm. Data are from a single experiment. Histograms show the mean ± SEM. *P < 0.05, **P < 0.01, ***P < 0.001 by 1-way ANOVA with Tukey’s multiple-comparison post hoc test.

To interrogate the functional role of prolymphangiogenic monocytes and monocyte-derived MΦs recruited to the site of filaria-parasitized lymphatics, we blocked CCR2^+^ monocyte recruitment following *Bm*L3 infection by administration of an anti-CCR2 ablating antibody (28). In a complementary approach, we reduced total phagocyte cell populations, including monocytes and MΦs, by local subcutaneous administration of clodronate encapsulated in liposomes (Figure 7A). Confirming treatment efficacy, both anti-CCR2 and clodronate liposome treatments delivered to filaria-infected mice successfully reduced circulating blood monocyte populations. Further, anti-CCR2 significantly reduced lymphatic-associated monocyte populations following infection (Figure 7, B and C). Following ablations of monocyte and total phagocyte populations, while remodeled lymphatics were still apparent, the magnitude of lymphatic insufficiency was significantly reduced, as demonstrated by reduced backflow of ICG following anti-CCR2 treatment (Figure 7, F and G) and dermal retention of EB (Figure 7H) following both anti-CCR2 and clodronate liposome treatments. Additionally, dermal lymphatic vessel dilation was significantly reduced following both anti-CCR2 and clodronate liposome treatments (Figure 7, I and J). These ablation experiments indicate a functional role for prolymphangiogenic monocyte populations, after recruitment from the blood to local parasitized lymphatics, in the development of filaria-associated lymphatic dysfunction.

### Second-generation tetracyclines target IL-4R–dependent MΦ lymphangiogenesis to ameliorate filarial lymphatic pathology.

**Figure 8:**
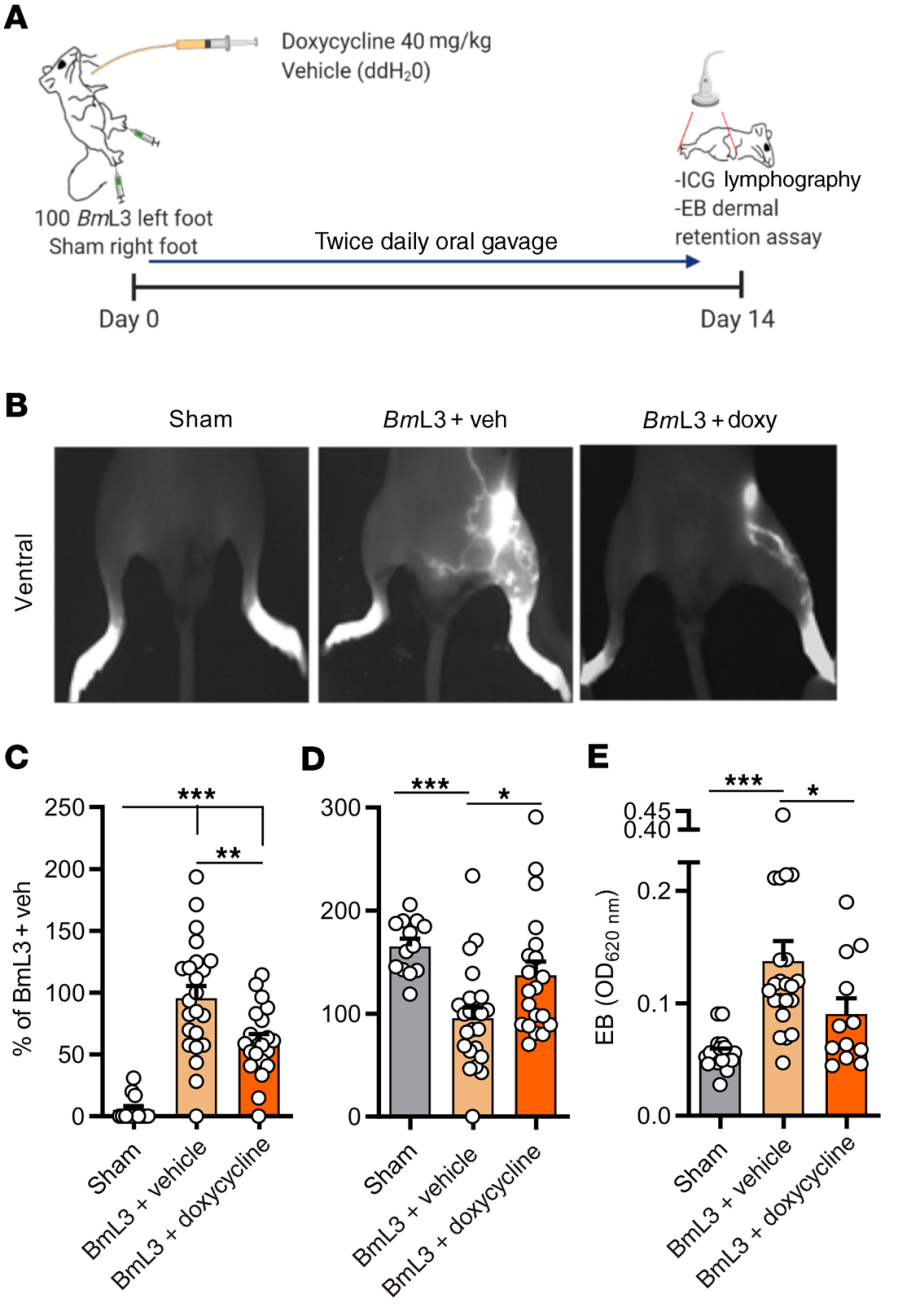
Doxycycline administration significantly ameliorates filarial lymphatic pathology. (**A**) Schematic of doxycycline intervention in *Bm*L3-infected C57BL/6J mice. (**B**) Representative images, (**C**) aberrant lymphatics, (**D**) hind-limb ICG dye retention in sham, *Bm*L3+vehicle, or *Bm*L3+doxycycline–treated mice, 14 dpi (*n =* 13 sham, *n =* 23 *Bm*L3+vehicle, *n =* 20 *Bm*L3+doxycycline). Data plotted as percentage change normalized to mean values of the *Bm*L3+vehicle control group in order to compare data pooled from independent experiments. (**E**) Evan’s blue dermal retention from left-hind-limb skin (*n =* 18 sham; *n =* 21 *Bm*L3+vehicle; *n =* 11 *Bm*L3+doxycycline). Data were pooled from 3 individual experiments. Histograms show the mean ± SEM. *P < 0.05, **P < 0.01, ***P < 0.001 by 1-way ANOVA with Tukey’s multiple-comparison post hoc test.

**Figure 9:**
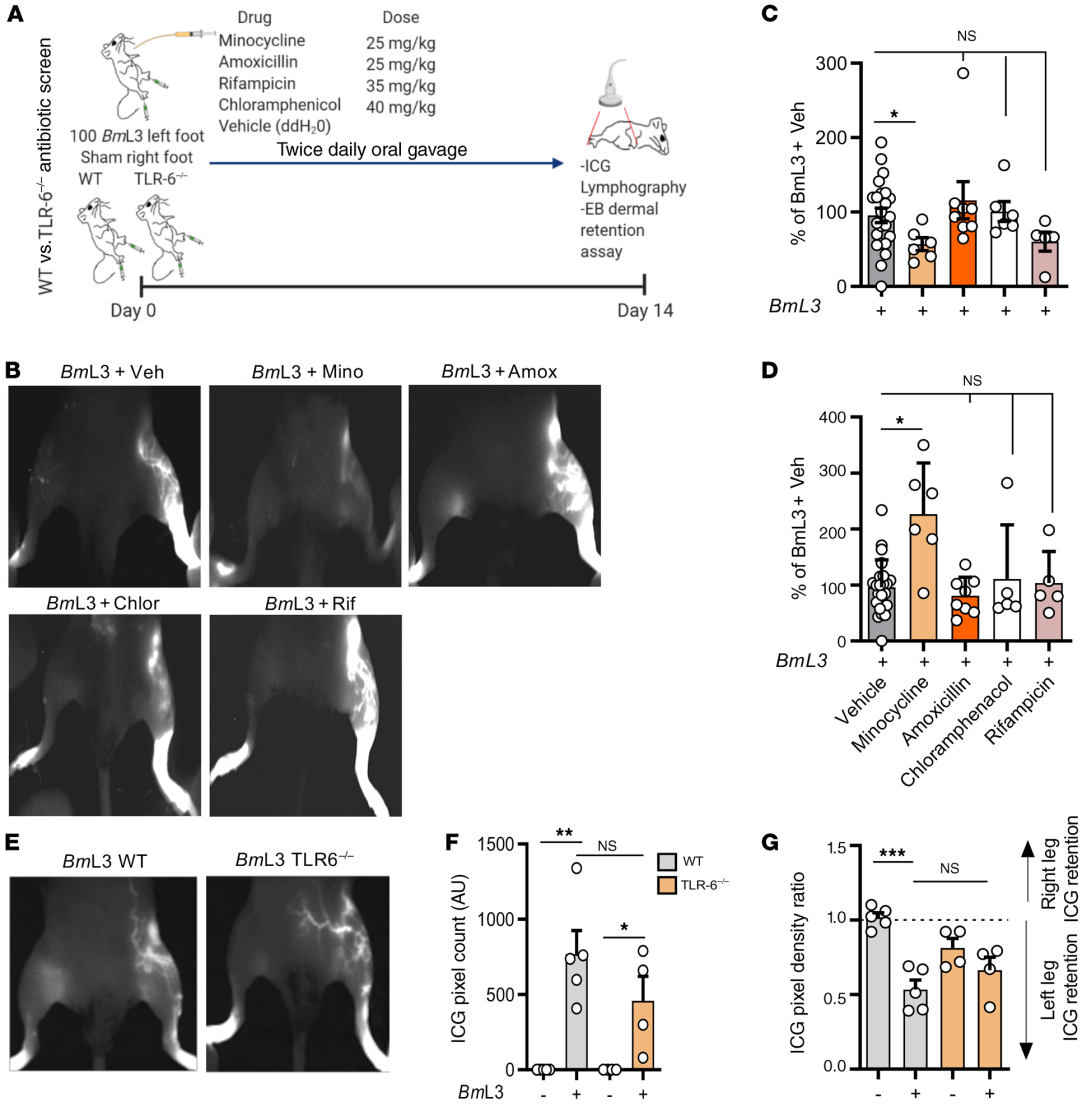
Doxycycline-mediated amelioration of filarial lymphatic pathology is independent of general antibiotic or anti-*Wolbachia* activity. (**A**) Schematic for antibiotic screen and Toll-like receptor 6 knockout (TLR6^–/–^) experiments in *Bm*L3-infected C57BL/6J mice. (**B**) Representative examples of PDE intravital imaging, (**C**) aberrant lymphatics, and (**D**) ICG hind-limb retention in *Bm*L3-infected mice treated twice daily with vehicle (*Bm*L3+Veh), minocycline (*Bm*L3+Mino), amoxicillin (*Bm*L3+Amox), chloramphenicol (*Bm*L3+Chlor), or rifampicin (*Bm*L3+Rif), 14 dpi (*n =* 23 *Bm*L3+Veh, *n =* 6 *Bm*L3+Mino, *n =* 8 *Bm*L3+Amox, *n =* 6 *Bm*L3+Chlor, *n =* 5 *Bm*L3+Rif). Data are percentage change normalized to mean of *Bm*L3+Veh mice in order to compare data pooled from independent experiments. (**E**) Representative examples of PDE intravital imaging, (**F**) aberrant lymphatics, and (**G**) ICG hind-limb retention in WT or TLR6^–/–^ *Bm*L3–infected mice or corresponding sham-infection controls, 14 dpi (*n =* 5 WT and TLR6^–/–^ sham; *n =* 4 WT+*Bm*L3; *n =* 6 TLR-6^–/–^+*Bm*L3). Data were pooled from 2 individual experiments (*Bm*L3+Amox in **C** and **D**) or a single experiment (*Bm*L3+Mino, *Bm*L3+Chlor, *Bm*L3+Rif groups in **C**, **D**, **F**, and **G**). Histograms show the mean ± SEM. *P < 0.05, **P < 0.01, ***P < 0.001 by 1-way ANOVA with Tukey’s multiple-comparison post hoc test. NS, not significant.

With previous work demonstrating antimorbidity efficacy of the second-generation tetracycline doxycycline in the treatment of filarial LE (7, 16, 29), we tested whether our preclinical filaria–lymphatic pathology model was responsive to oral doxycycline intervention. After 14 days of infection and cotreatment with a doxycycline regimen bioequivalent to human 200 mg daily oral dosing (ref. 30 and Figure 8A), mice exhibited significantly lower levels of both lymphatic remodeling (Figure 8, B and C) and lymphatic insufficiency compared with infected and vehicle control animals (Figure 8, D and E). We did not observe direct antifilarial efficacy at these treatment dose ranges against *B*. *malayi* developing larvae up to 14 days, ruling out a direct antiparasitic mode of action contributing toward reduced pathology (Supplemental Figure 6). Because the filarial endosymbiont *Wolbachia* is depleted by tetracyclines (30, 31) and can trigger innate inflammation via ligation of surface lipoproteins by Toll-like receptor 2 and 6 heterodimers (TLR2/6) (32), we investigated whether initiation of lymphatic pathology was influenced by *Wolbachia*. In addition, using the related second-generation antibiotic minocycline and a selection of different classes of antibiotics, we tested whether suppression of lymphatic pathology was a phenomenon unique to the tetracycline class or could be mediated by other antibiotics with anti-*Wolbachia* and/or broad-spectrum antibacterial activities (Figure 9A). We selected high-dose rifampicin as a broad-spectrum antibiotic with superior anti-*Wolbachia* activity compared with tetracyclines (33), as well as amoxicillin and chloramphenicol; both potent, broad-spectrum antibiotics lack significant anti-*Wolbachia* activity (34). Similar to effects observed with doxycycline, minocycline delivered at doses bioequivalent to 100 mg human oral exposures (30) led to significantly improved severity of lymphatic remodeling and insufficiency (Figure 9, B–D). Comparatively, none of the other administered broad-spectrum antibiotics — amoxicillin, chloramphenicol, or rifampicin — had any significant effect on either lymphatic remodeling or insufficiency following filarial lymphatic infection (Figure 9, B–D). Filaria-infected TLR6-deficient mice displayed no significant difference in either magnitude of lymphatic remodeling or lymphatic insufficiency compared with WT controls (Figure 9, E–G). Together, these data define a specific antimorbidity efficacy of second-generation tetracyclines in ameliorating filaria-induced lymphatic pathology, independently of general antibiotic or anti-*Wolbachia*–specific modes of action.

**Figure 10:**
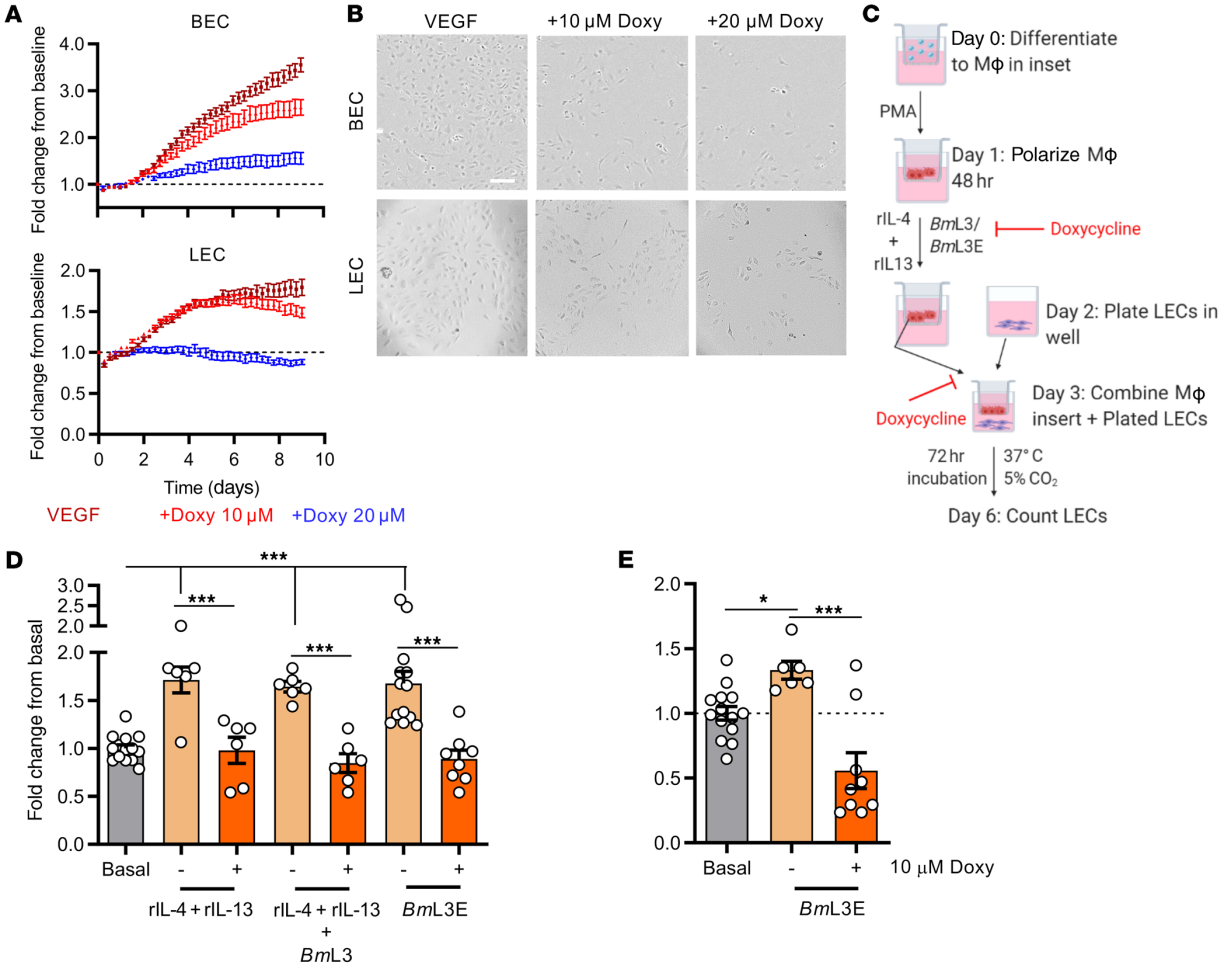
Doxycycline inhibits LEC proliferation directly and via impairment of type 2 or filaria-conditioned prolymphangiogenic MΦs. (**A**) BEC and LEC 9-day proliferation tracking following stimulation with 2 ng/mL VEGF, with or without 10 or 20 μM doxycycline. Data are fold-changes from initial BEC and LEC confluence. (**B**) Representative images of BEC and LEC confluence at endpoint. Scale bar: 500 μm. (**C**) Schematic of MΦ-LEC coculture indicating where doxycycline was added. (**D**) LEC enumeration following coculture with MΦs preconditioned with rIL-4+rIL-13, rIL-4+rIL-13+*Bm*L3, or *Bm*L3 extract (*Bm*L3E) with or without 10 μM doxycycline. (**E**) LEC enumeration following coculture with MΦs preconditioned with *Bm*L3E with or without 10 μM doxycycline. Histograms show the mean ± SEM. Data were derived from a single experiment (**A**) or pooled from 2 individual experiments (**D** and **E**). *P < 0.05, ***P < 0.001 by 1-way ANOVA with Tukey’s multiple-comparison post hoc test.

**Figure 11:**
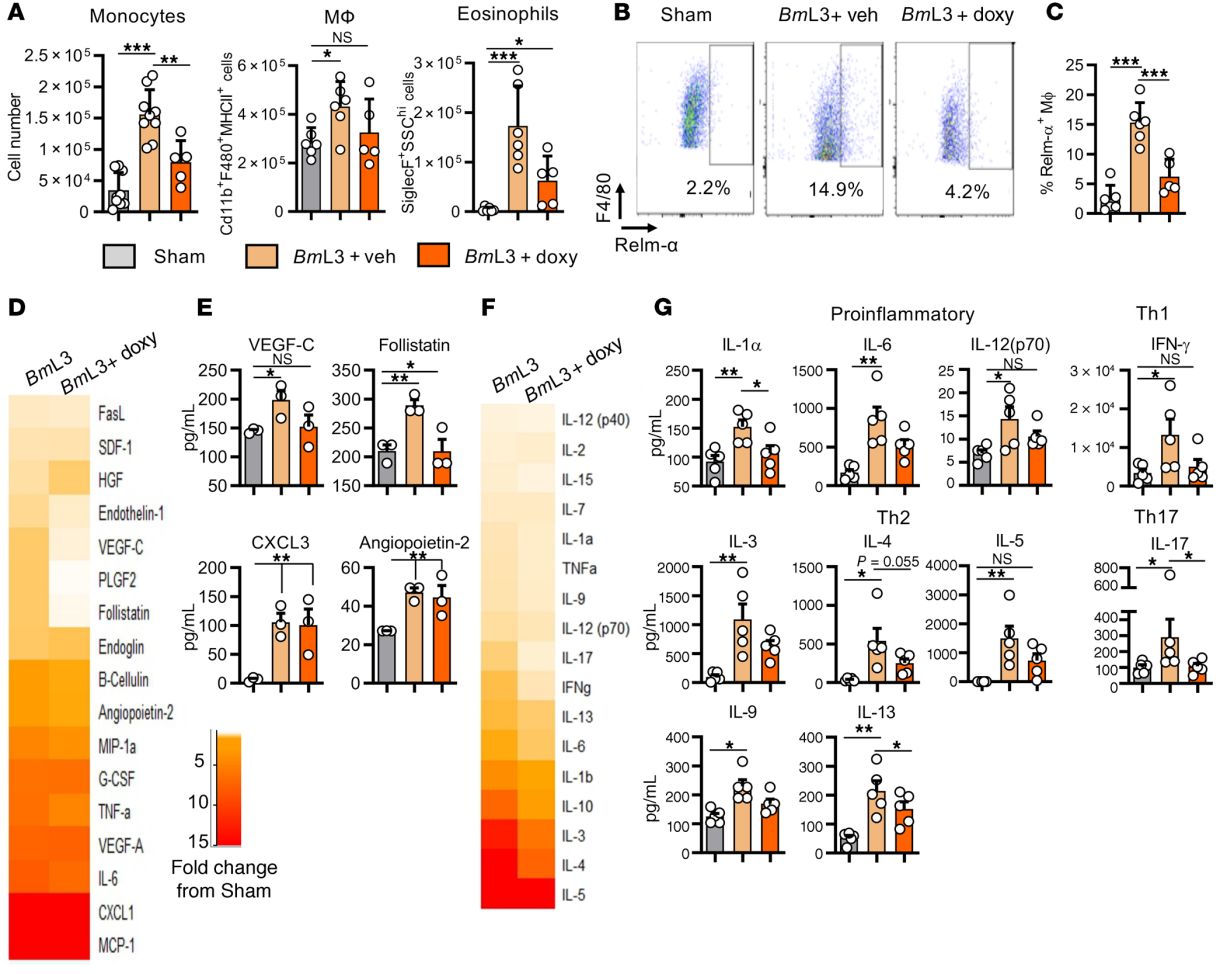
Doxycycline ameliorates filarial lymphatic pathology by modulation of IL-4R–dependent inflammatory lymphangiogenesis. (**A**) Immune cell populations from sdLNs and surrounding lymphatics from C57BL/6J sham- or *Bm*L3-infected mice treated with vehicle or 40 mg/kg doxycycline twice daily, 14 dpi. (**B**) Representative flow cytometry plots and (**C**) MФ expression of RELM-α (*n =* 6 sham and *Bm*L3+Veh, *n =* 5 *Bm*L3+doxycycline). Cells were gated on live CD11b^+^MHCII^+^F4/80^+^ cells. Data are RELM-α^+^ MΦs as a proportion of total MΦs. (**D**) Proteomic array of lymphangiogenic molecules in 72-hour cell cultures derived from sdLNs and lymphatic tissues, 14 dpi. Heatmap orange and red depict increasing fold-change compared with the mean of sham-infected mice. (**E**) Lymphangiogenic molecules attaining statistical significance; data plotted per mouse (*n =* 3 sham, *Bm*L3, and *Bm*L3+doxycycline). (**F**) Proteomic array of cytokine levels in splenocyte cultures 72 hours after polyclonal restimulation with anti-CD3/anti-CD28 antibodies. Heatmap orange and red depict increasing fold-change compared with the mean of sham-infected mice. (**G**) Cytokine concentrations attaining statistical significance, grouped under type of adaptive immune response; data plotted per mouse (*n =* 5 sham, *Bm*L3, and *Bm*L3+doxycycline). Histograms show the mean ± SEM. Data were pooled from 2 individual experiments (**A** and **C**) or derived from a single experiment (**E** and **G**). *P < 0.05, **P < 0.01, ***P < 0.001 by 1-way ANOVA with Tukey’s multiple-comparison post hoc test. NS, not significant.

We tested which facets of the type 2 inflammatory lymphangiogenic pathway induced by filarial infection were targeted by tetracyclines. We first investigated whether doxycycline could directly affect lymphangiogenesis in vitro. Growth assays, utilizing time-lapse microscopy to longitudinally quantify LEC or tissue-equivalent adult human dermal microvascular endothelial cell (blood endothelial cell; BEC) proliferation over 9 days were performed (Supplemental File 2). Treatment of LECs or BECs with 10 or 20 μM doxycycline impeded proliferation in response to a VEGF-A stimulus, in a dose-dependent manner (Figure 10, A and B, and Supplemental File 2). Similar effects were obtained with BECs and LECs treated with minocycline (Supplemental Figure 7). We then treated monocyte-derived MΦs with 10 μM doxycycline simultaneously during stimulation with live *Bm*L3, *Bm*L3 with type 2 cytokines, or *Bm*L3E. MΦs were washed before their transfer within Transwells onto LEC cultures to remove drug (Figure 10C). While rIL4+rIL13–, *Bm*L3+rIL4+rIL13–, and *Bm*L3E-pulsed MΦs mediated significant LEC proliferation, this affect was abolished by pretreatment with doxycycline (Figure 10D). Addition of 10 μM doxycycline to *Bm*L3E-pulsed MΦ and LEC cocultures also abrogated LEC proliferation (Figure 10E). No significant cytotoxicity was discerned when LEC or THP-1 MΦs were exposed to 10 or 20 μM doxycycline and LEC cultures responded to VEGF proliferating stimulus following removal of drug (Supplemental Figure 8). These in vitro data indicate that second-generation tetracyclines reversibly suppress VEGF-mediated lymphangiogenesis and, independently, the development of prolymphangiogenic monocyte–derived MΦs following filarial and/or type 2 cytokine stimulation. Using the filarial lymphatic pathology mouse model, we immunophenotyped lymphatic-associated myeloid cells from mice orally dosed with doxycycline compared with infection vehicle–dosed controls. Doxycycline-treated mice displayed significantly impeded monocyte recruitment compared with infection controls, while lymphatic-associated MΦ populations failed to expand (Figure 11A). Eosinophil levels in lymphatic tissues were also significantly reduced in infected mice following doxycycline treatment (Figure 11A). Doxycycline treatment also significantly blocked AAMΦ polarization as measured by reduced populations of RELM-α^+^ MΦs (Figure 11, B and C). We examined if this modified myeloid cell recruitment and reduced AAMΦ lymphangiogenic potential resulted in reduced local concentrations of the lymphangiogenic milieu. Ex vivo culture of single-cell suspensions prepared from sdLNs and adjacent lymphatic channels of filaria-infected mice treated with doxycycline demonstrated reductions in multiple lymphangiogenic secretions compared with infection controls (Figure 11D). Follistatin was significantly reduced, while VEGF-C secretions remained at sham-infection control levels (Figure 11E). We then examined whether the initial, predominant type 2 adaptive immune response important for mediating lymphatic pathology was perturbed by doxycycline. We assessed splenocyte recall assays to evaluate systemic immune responses. Doxycycline treatments modified numerous cytokines, compared with *Bm*L3 infection alone (Figure 11, F and G). Reductions in secretions of type 2 cytokines IL-3, IL-4, IL-9, and IL-5 were observed after doxycycline treatment in infected mice. Additionally, modified systemic type 1 (IFN-γ) and type 17 (IL-17) splenocyte secretions were recorded after doxycycline treatment. Further, general reductions in chemokine production, including those responsible for monocyte and macrophage activation (CXCL2, G-CSF), as well as the prolymphangiogenic growth factor VEGF-A were observed within splenocytes after doxycycline treatment (Supplemental Figure 9). Therefore, second-generation tetracyclines target multiple aspects of the type 2 inflammatory lymphangiogenic axis induced by filarial larval infection, as well as directly targeting lymphatic endothelial proliferation, to modify lymphatic filarial disease.

## Discussion

We reveal persistent lymphatic dilation, remodeling, and dermal backflow patterns in mice that emulate clinical lymphatic remodeling in both filarial and nonfilarial LE patients (14–17). Further, we record significant upregulation of the prolymphangiogenic circulating factors Ang-2, TNF-α, and VEGF-C, which are clinical serological markers of filariasis infection and LE pathology (29, 35, 36). Thus, we conclude that our preclinical model is representative of early lymphatic pathological changes in filariasis patients and a useful tool to interrogate the pathophysiology and therapeutic targeting of filarial disease.

Our model revealed that, surprisingly, abbreviated larval filarial infections, in as little as 6 days, could rapidly induce enduring lymphatic pathology without the necessity for establishment of chronic adult infections. It is currently not known whether such rapid pathology is evident in humans, as markers of adult filarial infection are typically utilized as selection criteria for study. However, a recent investigation has defined via lymphoscintigraphy that lymphatic pathology is evident in children as young as 5 years (37). Thus, we contend that frequent larval assaults transmitted by mosquito bites, that do not necessarily result in patent adult infections, may cause underappreciated lymphatic pathology in LF-endemic areas.

Strain-dependent magnitude of lymphatic remodeling, whereby BALB/c mice exhibited reduced pathology compared with C57BL/6J mice, reflects the relative vigor of sterilizing immunity against filarial infection between these 2 strains (38). Indeed, severity of LE in filariasis patients is associated with magnitude of CD4^+^ T cell immune responses to filarial antigen (20). In our model, local draining LN adaptive immune responses were polarized toward CD4^+^ T cell IL-4 and IL-13 secretion, suggesting an important role for type 2 sterilizing immune responses in induction of lymphatic dysfunction. We have defined eosinophil coordinated type 2 immune responses as critical to preventing *B*. *malayi* larval survival (22, 39). Lymphatic remodeling and dysfunction were reduced in SCID mice following filarial infection, demonstrating a requirement for adaptive immunity to induce early lymphatic dysfunction.

A limitation of our study was that while lymphatic pathology was rapidly induced, we did not observe overt LE in immunocompetent mice following a single infection event and up to 16 weeks follow-up. Further, we used a single high-dose infection (100 L3), whereas humans will be naturally exposed repetitively to low doses (typically <10 L3) in so-called trickle infections. Although dilation of *B*. *malayi* adult parasitized lymphatics and LE formation has been documented in *B*. *malayi*–susceptible T cell–immunodeficient mice (11, 13), reactivation of adaptive immunity during chronic infection time courses in aged mice was not scrutinized in these leaky lymphopenic models. Indeed, experimental immune reconstitution triggers a destructive, fibrotic, perilymphangitic pathology with myeloid-rich infiltrates in infected lymphatics coincident with immune-mediated killing of adult parasites (11). Further, in experimental infections of outbred feline and canine natural *Brugia* hosts, overt LE is associated with leukocytic intralymphatic obstructive thrombi and exacerbated by bacterial or fungal secondary infections (40, 41). In a susceptible ferret model of *B*. *malayi* infection, 6 trickle-dose inoculations over a 10-week period resulted in overt LE in 1 out of 4 animals tested (12). Thus, we suggest the immediate adaptive immune-dependent lymphatic pathology we detail is an early facet of a complex multifactorial process, likely requiring several chronic infection events within the limb lymphatic network and prime-boosting of type 2 immunity to culminate in pronounced lymphedematous disease.

In nonfilarial LE models, CD4^+^ T cell depletion reduces lymphatic pathology, while specific neutralization of type 2 cytokines IL-4 and IL-13 ameliorates edematous skin fibrosis (42, 43). Confirming the importance of type 2 immunity in filarial lymphatic pathology, IL-4R–deficient mice did not develop significant remodeling and were protected from lymphatic dysfunction after infection. IL-4R deficiency resulted in reductions in multiple circulating lymphangiogenic factors, notably VEGF-C and Ang-2, reduced monocyte/MΦ expansions within parasitized lymphatics, and prevention of MΦ alternative activation. We, and others, have previously described IL-4R–dependent alternative activation of serous cavity tissue MΦ populations in the context of filarial infection (22, 44). In oncology, dysregulated, tumor-derived stimuli polarize monocytes and MΦs into tumor-associated phenotypes, possessing similarities to AAMΦs, and resulting in increased tumor angiogenesis and lymphangiogenesis (45). In clinical filariasis, circulating monocytes with features of alternative activation have also been detected (46). We determined that lymphatic-associated monocytes and AAMΦs from parasitized tissues produced elevated VEGF-C, sALK-1, and prolactin, the 3 most upregulated prolymphangiogenic molecules in circulation following filarial infection, demonstrating that this cell lineage is a source of lymphangiogenic mediators at the site of filarial lymphatic pathology. Clinically, it has been shown that circulating blood mononuclear cells derived from filarial LE patients also demonstrate heightened VEGF-A/-C production upon ex vivo stimulation with either TLR or filarial antigens (47).

By serial depletion of CCR2^+^ monocytes or total phagocytes in vivo, we confirmed that temporal monocyte deficiency and impaired lymphatic recruitment alleviated lymphatic dysfunction and reduced lymphatic dilation. Similarly, CCR2^+^ monocyte recruitment has been demonstrated to mediate intestinal inflammatory lymphangiogenesis (48), whereas monocyte CD36 blockade prevents corneal lymphangiogenesis (49), suggesting a common mechanism in inflammatory lymphangiogenesis induction. We hypothesize that the gross local dilation in parasitized skin lymphangions impairs trafficking of solutes from proximal interstitial spaces during type 2 filarial inflammation. Lymphangion lumen dilation to the point of valve dysfunction has been proposed as a mechanism for lymphostasis in postsurgical LE (50). In filarial hydrocele pathology, gross honeycomb dilation of the supratesticular lymphatics correlates with circulating VEGF-A levels (15). As VEGF-A and VEGF-C both activate lymphatic endothelium via VEGFR1/2 and VEGFR3, respectively, our data support VEGF-A/-C–specific activation of the superficial lymphatics during filarial type 2 inflammation, delivered by recruited CCR2^+^ monocytes and their subsequent differentiation into AAMΦs. However, we also identified circulating and monocyte-specific production of other lymphangiogenic factors, namely sALK-1 and prolactin, while another lymphangiogenic factor, Ang-2, which was IL-4R type 2 dependent in circulation, was not produced by the monocyte/MΦ lineage within parasitized lymphatics. This suggests additional lymphangiogenic factors contribute to remodeling events during initiation of type 2 filarial inflammation within sdLNs. The relative functional roles of these multiple growth factors need investigating to determine whether targeted antiangiogenics may be of therapeutic benefit in filarial LE.

In our human cell coculture system, polarization of monocyte-derived MΦs with type 2 cytokines resulted in a MΦ phenotype able to induce LEC proliferation. However, live filarial larvae or their products could also induce an MΦ phenotype without additional type 2 cytokine help. Type 2 or filaria-polarized monocyte-derived MΦs in vitro produced increased secretions of VEGF-A/-C, follistatin, and HGF. Filaria-specific activation of human CD14^+^ monocytes has been previously demonstrated to induce prolymphangiogenic VEGF-A secretions (9). Thus, local patrolling CD14^+^ monocyte populations in the lymphatics may also be able to facilitate localized lymphatic dilations in the immediate vicinity of invading larvae in response to larval secretions. This may facilitate larval migrations through lymphatics and would occur prior to initiation of type 2 immunity, resulting in the recruitment of inflammatory monocytes, their differentiation into AAMΦs, and resultant augmented and widespread lymphatic pathology.

Prior clinical research has promoted an antipathological role of 6-week 200 mg/day doxycycline treatment in ameliorating filarial LE pathologies (7, 16, 29, 51). Reduced circulating VEGF-A/-C was observed in these studies, strengthening the hypothesis that chronic lymphatic remodeling supports development and maintenance of filarial LE (7, 16, 29). The mechanism by which doxycycline mediates antimorbidity effects in filariasis is difficult to determine in the clinic, due to its curative activity via targeting filarial *Wolbachia* (52), and its broad-spectrum antibiotic properties that reduce secondary skin bacterial infections and cellulitis complications (53). Further, *Wolbachia* can directly activate classical inflammatory processes upon liberation from filarial tissues (32) and have been identified as mediators of systemic adverse reactions in LF patients after filaricidal treatment (54, 55). Therefore, *Wolbachia* may contribute to filarial LE via triggering classical inflammation (56) and doxycycline may prevent this disease pathway. Upon characterizing a type 2 inflammatory response causal in inducing filarial lymphatic pathology, we exploited our model systems to investigate the mode of action by which second-generation tetracyclines ameliorate filarial lymphatic disease. First, we established that both doxycycline and the related second-generation tetracycline, minocycline, are directly antilymphangiogenic, blocking LEC proliferation in response to VEGF stimuli. These data confirm earlier reports that doxycycline directly modifies VEGF-C–induced LEC proliferation by interrupting phosphorylation of phosphoinositide 3 kinase (PI3K), α-serine/threonine protein kinase (AKT1), and endothelial nitric oxide synthase (eNOS) signaling (57). We also determined that the suppressive effect of doxycycline extends to inhibiting LEC proliferation mediated by IL-4/-13 or filaria-conditioned proangiogenic MΦs. The antiangiogenic pharmacological activity of doxycycline or minocycline achieved in vitro, at 10 or 20 μM, was at or slightly higher than typical clinical peak-plasma concentrations. However, concentrations of doxycycline, following 14-day dosing in the skin, are known to accumulate 3-fold more than measured in circulation (58). This suggests our effective dose levels reflect local concentrations experienced within and surrounding superficial lymphatics.

Antilymphangiogenic activities of doxycycline and minocycline were reproducible in vivo, whereby oral dosing of mice with human bioequivalent regimens (30) significantly reduced the magnitude of lymphatic remodeling and dysfunction induced by filarial infection. We determined that this antipathological mechanism was tetracycline specific and unrelated to broad-spectrum antibiotic or anti-*Wolbachia* efficacies. Lack of evidence for *Wolbachia* in lymphatic pathology induction in our larval model probably reflects low *Wolbachia* titers in infectious stage *B*. *malayi* and does not necessarily preclude a role for higher titers of *Wolbachia*, liberated upon death of more mature filariae in parasitized lymphatics, augmenting LE pathology development in vivo. The skewed, local type 2 inflammation observed in our mouse model also reflects low *Wolbachia* exposure during initial immune priming, as we previously demonstrated that type 2 T cell polarization by filarial extract becomes modified toward a mixed type 1 and type 2 T cell response by relative abundance of *Wolbachia* products (32).

Doxycycline modified the type 2 recruited monocyte/AAMΦ pathway of lymphatic pathology at multiple points in vivo. Thus, we demonstrate that doxycycline has wide-ranging immunosuppressive and antiinflammatory activities in modulating filaria-induced type 2 inflammatory lymphangiogenesis. As doxycycline directly perturbed prolymphangiogenic MΦs in response to type 2 or filaria-specific stimuli in vitro, this provides evidence of a specific targeted effect at the level of MΦs. Doxycycline has previously been shown to suppress IL-4/-13–dependent alternative activation of monocyte-derived MΦs, with concomitant impairment in MΦ-induced angiogenesis (59). The likely multifaceted mechanisms by which second-generation tetracyclines cause such wide-ranging antilymphangiogenic, antiinflammatory, and immunosuppressive effects on mammalian cells to stymie filarial type 2 lymphatic pathogenesis require further detailed investigations. An assumed mode of doxycycline-mediated antiangiogenic activity in vivo has been via targeted inhibition of matrix metalloproteinases (MMPs) to prevent extracellular matrix degradation necessary for neovascularization (60, 61). One alternative, emerging mechanism is that doxycycline suppresses mammalian mitochondrial protein synthesis, thus shifting cellular metabolism toward glycolysis and slowing the cell proliferative rate (62). Finally, a recent study demonstrates that calcium signaling is relevant in VEGF-A–induced angiogenesis (63). Because doxycycline is a known calcium ion chelator, antiangiogenic and more widespread antiproliferative effects of the drug could be mediated by attenuating multiple calcium-dependent, second messenger signaling pathways. Certainly, the T cell antiproliferative activity of doxycycline can be overcome by addition of exogenous calcium (64).

As with current indications in the treatment of rheumatoid arthritis or rosacea (65), we found that the mode of action of second-generation tetracyclines in mediating antipathological efficacy in filariasis is via immunosuppressant/antiinflammatory activities. However, akin to the dual mode of action considered important in the treatment of acne (65), we do not discount that second-generation tetracyclines are also beneficial to filarial LE patients by resolving secondary bacterial infections, preventing ADLA episodes. Lipophilicity and dermal accumulation of second-generation tetracyclines may be important physiochemical features contributing to a long tail of antipathological activities in superficial lymphatics and local sdLNs. Because minocycline is a more lipophilic antibiotic compared with doxycycline (30), it may be a clinically superior treatment for filarial LE, warranting comparative clinical assessment, while newly approved formulations of minocycline (66) for the treatment of skin complaints warrant clinical assessment of antipathological effects in filarial LE patients.

Because sterile postsurgical LE has been clearly linked with inflammation and leukotriene production (67), doxycycline may be of therapeutic benefit in the treatment of nonfilarial LE of inflammatory origin, especially where cellulitis complications contribute to disease etiology.

Potential limitations of the deployment of oral second-generation tetracyclines as antimorbidity therapy for filarial LE include the potential for gastrointestinal side effects, development of photosensitivity, and contraindications during pregnancy and for young children. However, large-scale implementation trials of doxycycline treatment as a cure for filariasis in over 13,000 African participants have determined greater than 90% adherence to treatment and phase II trials have only reported infrequent and generally mild adverse effects during 6-week therapy (68). Large-scale, multicenter trials are currently commencing to evaluate doxycycline as an antimorbidity therapy for filarial LE (69). Future clinical trials should also address dose duration and frequency, comparative efficacy of doxycycline versus minocycline, and whether addition of affordable nonsteroidal antiinflammatory drugs, such as ketoprofen, which is currently undergoing clinical assessment for the treatment of postsurgery LE (70), may be of added benefit, including in contraindicated groups.

In conclusion, our preclinical research establishes the mode of action of second-generation tetracyclines as antimorbidity drugs in the therapy of filarial LE. These findings support the onward clinical evaluation of these affordable, readily available, and safe treatments for LE of filarial origin and potentially for other LE associated with chronic inflammation.

## Methods

### Study design.

Group sizes of animal experiments were determined using appropriate sample size calculations to power a study greater than 80%. Data were pooled from repeat experiments where done. Mice were randomized into infection/intervention groups by ID number. Dosing and interventions were done in a nonblinded manner. Image-based readouts were blinded prior to analysis.

### Experimental animals.

Laboratory animals were maintained in specific pathogen–free facilities at The Biomedical Services Unit, University of Liverpool. Mongolian gerbils and BALB/c/C57BL/6J IL-4Rα^–/–^, C57BL/6J Prox-1^GFP^, and C57BL/6J TLR6^–/–^ mice were bred in house. Mongolian gerbils were originally purchased from Charles River. BALB/c IL-4Rα^–/–^ mice were originally purchased from The Jackson Laboratory. C57BL/6J IL-4rα^–/–^ mice were originally gifted by Cecile Benezech (The University of Edinburgh, United Kingdom). FVB/N-Crl:CD1(ICR) Prox-1^GFP^ mice were provided by Young-Kwon Hong, University of Southern California, before being backcrossed onto the C57BL/6J background for 7 successive generations. C57BL/6J TLR6^–/–^ mice were originally gifted by Shizuo Akira (Osaka University, Japan). Male BALB/c, C57BL/6J WT, and CB.17 SCID mice were purchased from Charles River. All mice were 6–12 weeks old at the start of procedures. Gerbils were infected between 8 and 12 weeks of age. Males were used in this study.

### Parasite life cycle and maintenance.

*B*. *malayi* life cycle was maintained in mosquitoes and Mongolian gerbils as previously described (31). Briefly, microfilariae (mf) from gerbils infected more than 12 weeks were collected via peritoneal catheterization. Purified and enumerated mf were mixed with heparinized human blood to 15–20,000 mf/mL and artificial membrane feeder (Hemotek) fed to female *Aedes aegypti* mosquitoes. After 14 days, infective *Bm*L3 were collected from infected mosquitoes by crushing and Baermann’s filtration.

### Leg pathology model experimental infection.

Mice were inoculated with 100 *Bm*L3 s.c., split between the top of the left hind foot and caudal to the left knee. Sham-infected mice received equal volumes of sterile RPMI 1640.

### Intravital NIR imaging of lymphatics.

NIR imaging was adapted from techniques previously described (17). Briefly, anesthetized mice were administered 20-μL s.c. injections of 1 mg/mL ICG (MilliporeSigma) onto the top of the left and right hind feet. Lymphatic drainage was monitored using a photodynamic eye (PDE) NIR optical imaging device (Hamamatsu Photonics) to track NIR signals. Mice were imaged from 4 viewpoints: dorsal, ventral, left, and right. Movies (720 × 480 at 60 fps; 3 minutes per mouse) were recorded using an EasyCap DC60 USB Video Capture Card Adapter (Softonic) that converted footage to ImageJ software (NIH). Still images (720 × 480) were used in downstream analyses. For more information see Supplemental Methods.

### EB dermal retention assay.

A modified Miles assay was utilized whereby mice were administered s.c. injections of 10 μL 1% EB (MilliporeSigma) w/v in sterile Dulbecco’s PBS (DPBS) (MilliporeSigma) on top of the infected hind foot. After 20 minutes, mice were euthanized and left hind leg skin excised between the knee and ankle joint, transferred to 1 mL of DPBS, and incubated 20 minutes. Absorbance was read at 620 nm on a Varioskan plate spectrometer (Bio-Rad).

### Fluorescence microscopy.

Skin samples from C57BL/6J Prox-1^GFP^ mice were dissected from areas of aberrant lymphatics (equivalent areas used in sham control mice). Lymphatic vessels were visualized using Prox-1^GFP^ epifluorescence under a fluorescence stereo-dissecting microscope with an eGFP filter (Leica Microsystems). Between 15 and 30 images were taken per mouse, blinded, and lymphatic channels measured for aperture in ImageJ. All image measurements were pooled per mouse to calculate average lymphatic widths.

*Bm*L3 were washed before incubation with 50 μM Alexa Fluor 546 NHS ester (succinimidyl ester) (Thermo Fisher Scientific) in Fluorobrite DMEM (Thermo Fisher Scientific) for 2 hours. C57BL/6J Prox-1^GFP^ transgenic mice were injected with 400 fluorescent *Bm*L3 as described above. After 3 hours and 1–6 dpi in mice, areas of subcutaneous tissues where lymphatic remodeling occurs were imaged as above (DsRed and eGFP filters).

### Lymphoid, lymphatic, splenic, and blood single-cell preparations.

Cardiac blood was collected into heparinized tubes (Starstedt), centrifuged, plasma harvested, and stored at –80°C for downstream analyses. In blood immunophenotyping experiments, red blood cells were depleted using RBC lysis buffer (Biolegend), resuspended in DPBS with 5% fetal bovine serum (FBS) and 2 mM EDTA (FACS buffer). Spleens or popliteal, iliac, and subiliac LNs with surrounding lymphatic collecting vessels were collected and a single-cell suspensions made by maceration through a 40-μm cell sieve (MilliporeSigma). Resultant cell suspensions were centrifuged, resuspended in RPMI 1640 or FACS buffer, and enumerated.

### Splenocyte and LN cell recall assays.

LN cells and splenocytes were plated at 2.5 × 10^5^/well: splenocytes into wells previously coated with 1.25 μg/mL anti-CD3 antibody followed by the addition of 2 μg/mL anti-CD28 antibody (Biolegend). LN cells received no ex vivo stimulation. All cells were incubated for 72 hours at 37°C and 5% CO_2_ and subsequent supernatants frozen at –20°C.

### Multiplex protein array analysis.

Multiplex immunoassays of 25 growth factors or 32 cytokines/chemokines (Mouse Angiogenesis/Growth Factor/Mouse Cytokine/Chemokine Magnetic Bead Panels, Merck) were undertaken on plasma or restimulated splenocyte/LN cell cultures, following the manufacturer’s protocol. Plates were read on the Bioplex 200 system (Bio-Rad) and data analyzed using Luminex XPONENT software.

### Flow cytometry.

Single-cell suspensions were FcR blocked before staining with viability dye and specific fluorescently labeled antibodies (Supplemental Table 1), as previously described (22). For intracellular cytokine experiments, sdLN suspensions were stimulated for 5 hours in Cell Stimulation Cocktail (eBioscience), followed by anti-CD4 and intracellular cytokine staining. Data were acquired on an LSRII flow cytometer (BD Biosciences) and analyzed using FlowJo software (BD Biosciences) (Supplemental Figure 4).

### FACS and cell secretion assays.

Following surface staining, cell populations (Supplemental Figure 4) were sorted using a FACSAria II (BD Biosciences) to 95% or greater purity into ice-cold DPBS with 40% FBS and 2 mM EDTA. Purified B and T cells were plated at 1 × 10^6^ cells/250 μL, while monocytes and macrophages were plated at 2.5 × 10^5^ cells/100 μL into 96-well plates (STARLAB). Cells were incubated at 37°C and 5% CO_2_ for 72 hours and collected supernatants frozen at –80°C.

### Cell culture.

Primary (adult) human dermal lymphatic microvascular endothelial cells (HMVEC-dLyAd; LECs) and human dermal microvascular endothelial cells (HMVEC-dAd; BECs) were purchased from Lonza and passaged in an Endothelial Growth Medium-2 Bullet kit (EGM-2) (Lonza). THP-1 monocytes (ECACC, Public Health England) were passaged in RPMI 1640 (MilliporeSigma) supplemented with 10% FBS (MilliporeSigma), 100 IU/mL penicillin/streptomycin (MilliporeSigma), and 2.5 mg/L amphotericin B (MilliporeSigma). All cells were maintained at 37°C and 5% CO_2_.

### BmL3E.

Batches of 1000–2000 *Bm*L3 were washed, resuspended in E-toxate water (MilliporeSigma), and extracts prepared as previously described (32), before storage at –20°C.

### MΦ/LEC coculture assay.

THP-1 monocytes were plated in 12-well Transwell inserts at 1 × 10^6^ cells/well and differentiated into MΦs using 10 ng/mL PMA (MilliporeSigma) for 24 hours. Inserts were washed and stimulated with indicated combinations of 10 μg/mL *Bm*L3E, 10 *Bm*L3, 10 ng/mL human rIL-4+rIL-13 or rIFN-γ (all Peprotech), 10 μM doxycycline, or 20% EGM-2/80% endothelial basal media mix media only, for 48 hours. LECs were seeded separately on 12-well plates at 4 × 10^4^ cells/well. Following 48-hour stimulation, inserts and LEC wells were washed, combined, and incubated for 72 hours at 37°C and 5% CO_2_. LECs were enumerated following harvesting from plates by microscopy.

### LEC/BEC proliferation assays.

LECs/BECs were plated at 2 × 10^5^ cells/well in 96-well plates, stimulated with 2 ng/mL VEGF165 (VEGF) (Lonza) with or without 10 or 20 μM doxycycline or minocycline (MilliporeSigma) and maintained at 37°C and 5% CO_2_ for 10 days. Proliferation was quantified longitudinally using the Incucyte live-cell imaging platform with images taken hourly and results plotted as fold change from confluence at hour 0.

### CCR2 and clodronate liposome monocyte/MΦ depletion experiments.

Following infection, mice were administered either 20 μg MC-21 rat anti–mouse CCR2 depleting antibody (Matthias Mack, Regensburg University; ref. 28) i.p., daily, or 2.5 mg/mL clodronate liposome suspensions (Liposoma) s.c. at *Bm*L3 infection sites every 3 days. Treatment was undertaken for 6 days.

### Antibiotic screens.

Infected mice were randomized into groups and administered the following twice per day: 40 mg/kg doxycycline, 25 mg/kg minocycline, 25 mg/kg amoxicillin, 35 mg/kg rifampicin, 40 mg/kg chloramphenicol (all MilliporeSigma), or ddH_2_O vehicle control via oral gavage for 14 days.

### Statistics.

All continuous data were tested for normal distribution using the Kolmogorov-Smirnoff test. Where data were normally distributed, a 2-tailed independent Student’s *t* test (2 groups) or 1-way ANOVA with Tukey’s post hoc comparisons test (>2 groups) was used to test for significant differences. Where data were found to be not normally distributed, a log transformation was first attempted. If data remained nonparametric, a 2-tailed Mann-Whitney *U* test (2 groups) or Kruskal-Wallis with Dunn’s post hoc multiple-comparison test (>2 groups) was utilized to test for significant differences between groups. The mean ± SEM are reported in all data unless otherwise stated. A *P* value less than 0.05 was considered significant. Significance is indicated as *P < 0.05, **P < 0.01, ***P < 0.001, ****P < 0.0001.

### Study approval.

All rodent experimental procedures were approved by the Animal Welfare Committee of University of Liverpool and The Animal Welfare and Ethics Review Board of Liverpool School of Tropical Medicine (LSTM) and carried out in accordance with The Use of Animals in Scientific Procedures Act.

## Author contributions

JFS, SDC, MJT, and JDT designed the research studies. JFS, SDC, AEM, NP, JA, and AS conducted the research. JFS and SDC analyzed the data. SSM, MM, YKH, MJT, and JDT provided reagents and resources. JFS and JDT wrote the manuscript.

## Supplementary Material

Supplemental data

Supplemental Data Set 1

Supplemental Data Set 2

Supplemental Video 1

## Version 1 01/12/2021

In-Press Preview

## Version 2 03/01/2021

Print issue publication
