# Supplementary material for: Tetracyclines improve experimental lymphatic filariasis pathology by disrupting interleukin-4 receptor–mediated lymphangiogenesis
Source: J Clin Invest. 2021 Mar 1;131(5):e140853. doi: 10.1172/JCI140853 (PMC7919730; doi:10.1172/JCI140853)
Supplement: Supplemental data [file jci-131-140853-s241.pdf]

Tetracyclines improve experimental lymphatic filariasis pathology by disrupting interleukin-4 receptor-mediated lymphangiogenesis

Supplemental Information

Julio Furlong-Silva, Stephen D Cross, Amy E Marriott, Nicolas Pionnier, John Archer, Andrew Steven, Stefan Schulte Merker, Matthias Mack, Young-Kwon Hong, Mark J Taylor and Joseph D Turner*

*email: joseph.turner@lstmed.ac.uk. Telephone: +44 (0)151 7053119

Supplemental Figure 1

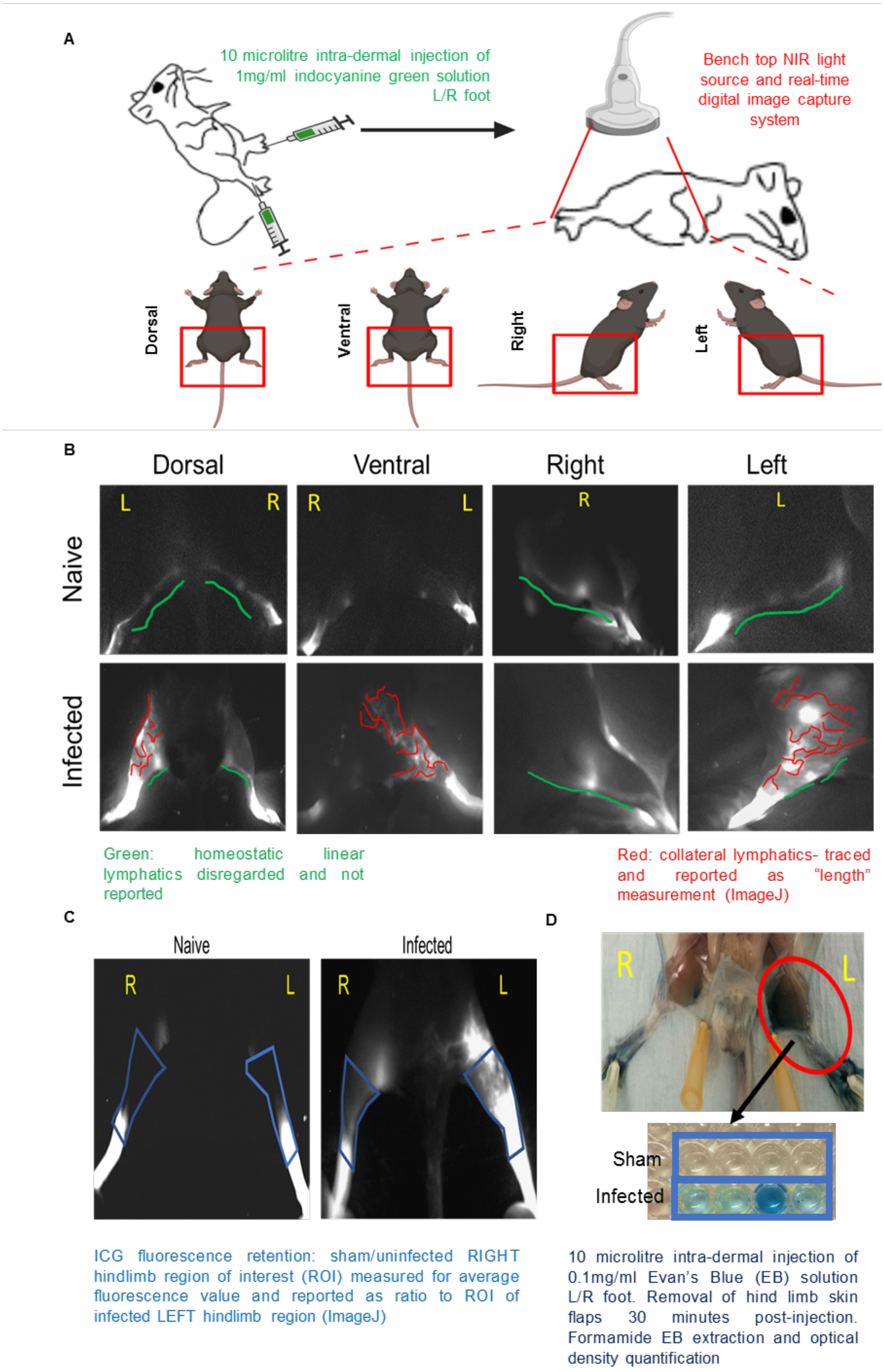

Supplemental Figure 1 *Development and quantification of an inflammatory hind limb infection model of Brugia malayi filarial infection*

A) Schematic representation of intravital imaging and imaged viewpoints used to quantify lymphatic remodelling and lymphatic insufficiency **B)** Method of quantifying lymphatics. Normal lymphatic flow (highlighted in green) are disregarded, while any lymphatic channels appearing outside of normal homeostatic flow patterns (highlighted in red) are traced and reported as length measurements in ImageJ. The sum of traced length measurements is reported as a quantitative readout of total remodelled lymphatics **C)** Method of quantifying lymphatic insufficiency. Region of interest (ROI; blue polygon selections) are drawn from ankle to below knee on both uninfected right hind-limb and sham/*Bm*L3 infected left hind-limb. Retention of ICG dye in the ROI corresponds to in higher infrared fluorescence (white). **D)** Representative images of modified Miles Assay to measure dermal retention of Evan's Blue (EB) as a corroboratory measure of lymphatic insufficiency.

Supplemental Figure 2

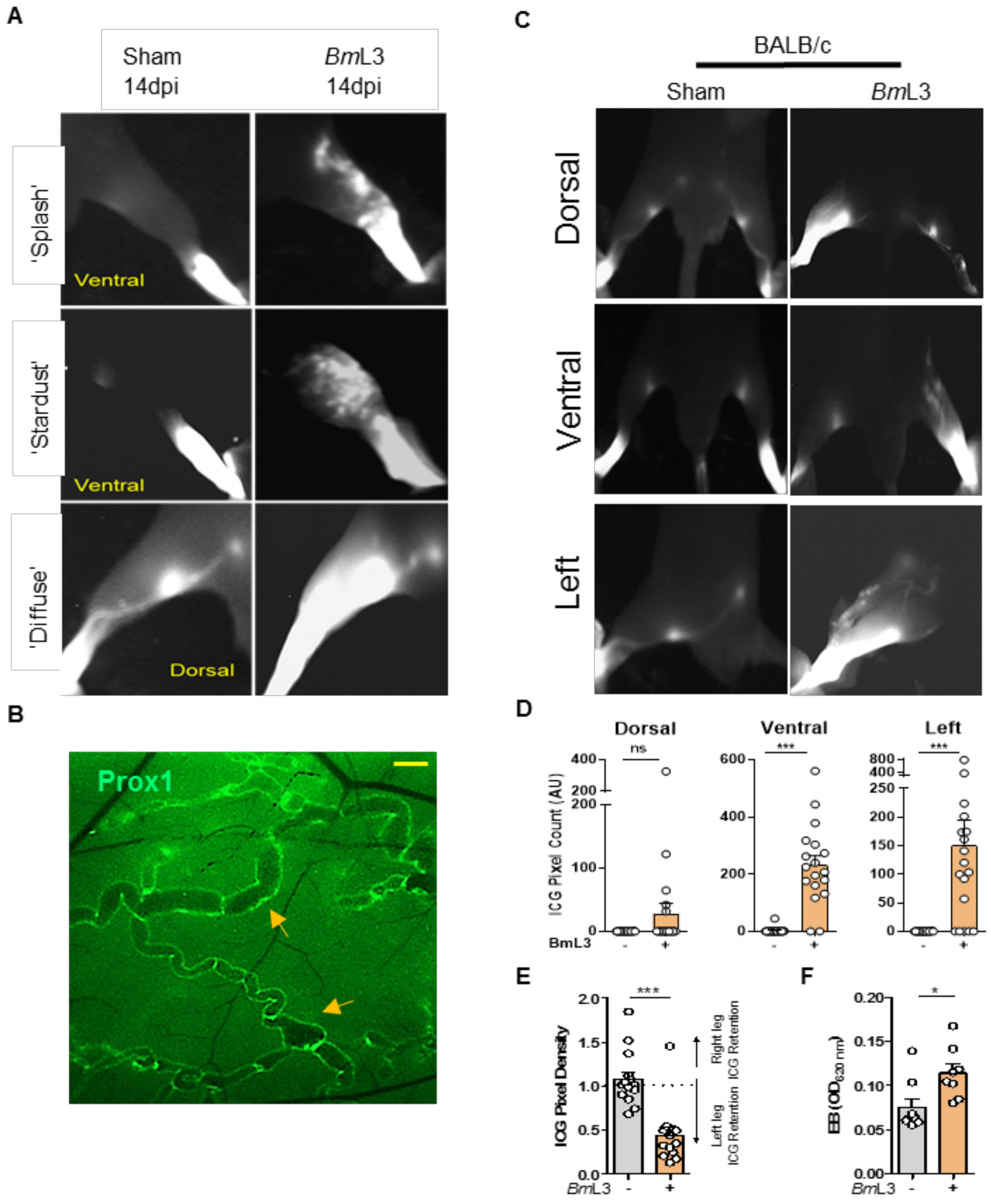

Supplemental Figure 2 *Bm*L3 *induced pathological lymphatic backflow, dysfunction and tortuous collateral lymphatic characterisation in C57BL/6J and BALB/c mice.*

A Representative PDE images of pathological backflow patterns as previously described *(17)*, following 14d *Bm*L3 infection in C57BL/6J mice. **B)** Representative image from epifluorescence microscopy of *Bm*L3 infected Prox-1^{GFP+} C57BL/6J mice, 14dpi, viewing the dermal lymphatic vasculature, in common areas of lymphatic remodelling as imaged using PDE. Arrows mark examples of tortuous collateral lymphatic channels observed in BmL3 infected mouse cohorts. Scale bar= 200µm **C)** Representative PDE imaging from Sham/*Bm*L3 infected BALB/c mice **D)** Quantified aberrant lymphatics and **E)** lymphatic insufficiency from PDE imaging between sham(-) and *Bm*L3(+) infected BALB/c mice, 14dpi (n=20 Sham, n=18 *Bm*L3) **F)** EB accumulation in left hind-limb dermis between sham and *Bm*L3 infected BALB/c mice (n=6 sham, n=8 *Bm*L3). Bars are mean±SEM pooled from 3 independent experiments (D-E) or a single experiment (F). Statistical significance is indicated as *=P<0.05, **=P<0.01, ***=P<0.001, ns=not significant, one-way ANOVA with Tukey's multiple comparison post-hoc test.

Supplemental Figure 3

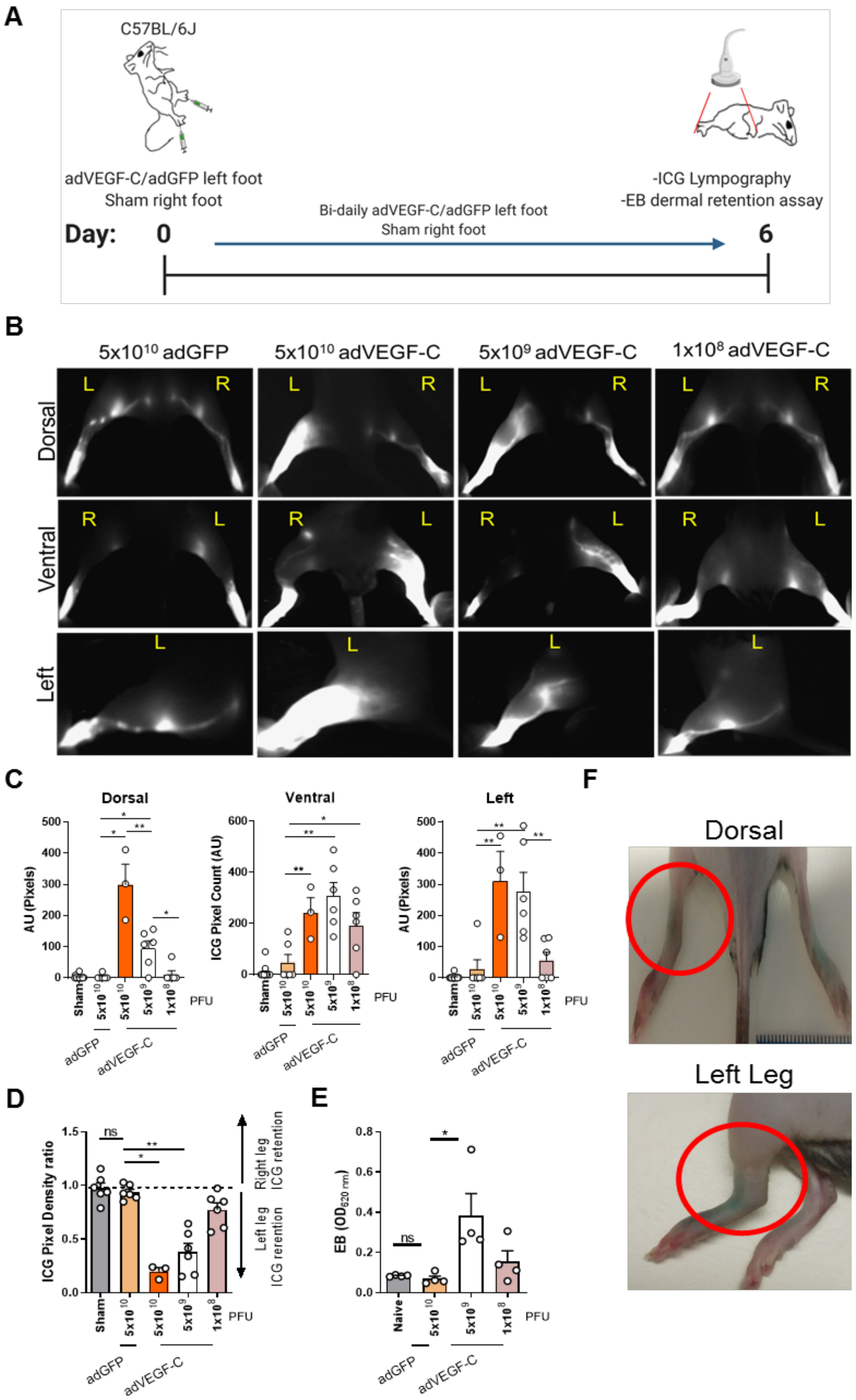

Supplemental Figure 3 *Local upregulation of VEGF-C signalling is sufficient to drive significant lymphatic remodelling and insufficiency in naïve mice*

A) Schematic of adenoviral infection model involving bi-daily administration of VEGFC (adVEGFC) overexpressing or GFP (adGFP) adenoviruses in varying dosages administered to the left hindlimb foot of naïve C57BL/6J mice subcutaneously **B)** Representative images from PDE intravital imaging 6dpi **C)** Quantified aberrant lymphatics and **D)** Quantified ICG retention from PDE imaging between groups. **E)** Comparison of Evan's blue left hind limb dermal retention from excised lower left hindlimb skin between groups (Sham data from Figure 1D; N=3 $5x10^{10}$ adVEGFC; N=5 $5x10^{9}$ adVEGFC; N=5 $1x10^{8}$ adVEGFC, N=5 $5x10^{10}$ adGFP). **F)** Representative images demonstrating visible lymphoedema in left hindlimb of mice administered $5x10^{10}$ PFU adVEGFC. Red circles highlight areas of oedema with opposite right hind-limb shown for reference. Bars represent the mean ±SEM. Data is from 1 experiment. Significance is indicated as *=P<0.05, **=P<0.01, ***=P<0.001, ns=not significant derived from a one-way ANOVA with Tukey's multiple comparisons post-hoc test between marked groups.

Supplemental Figure 4

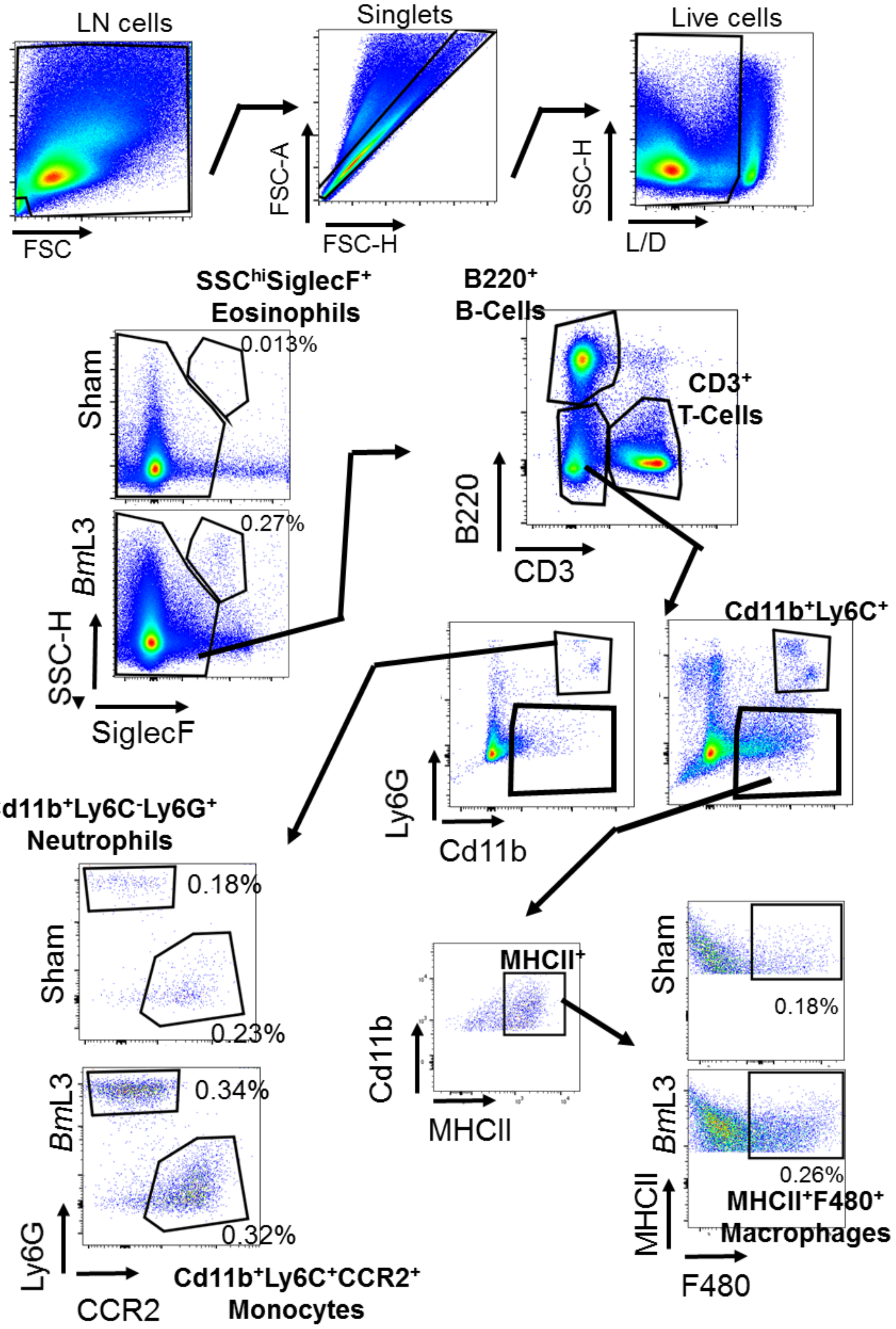

***Supplemental Figure 4** Gating strategy utilised for immunophenotyping experiments*

Supplemental Figure 5

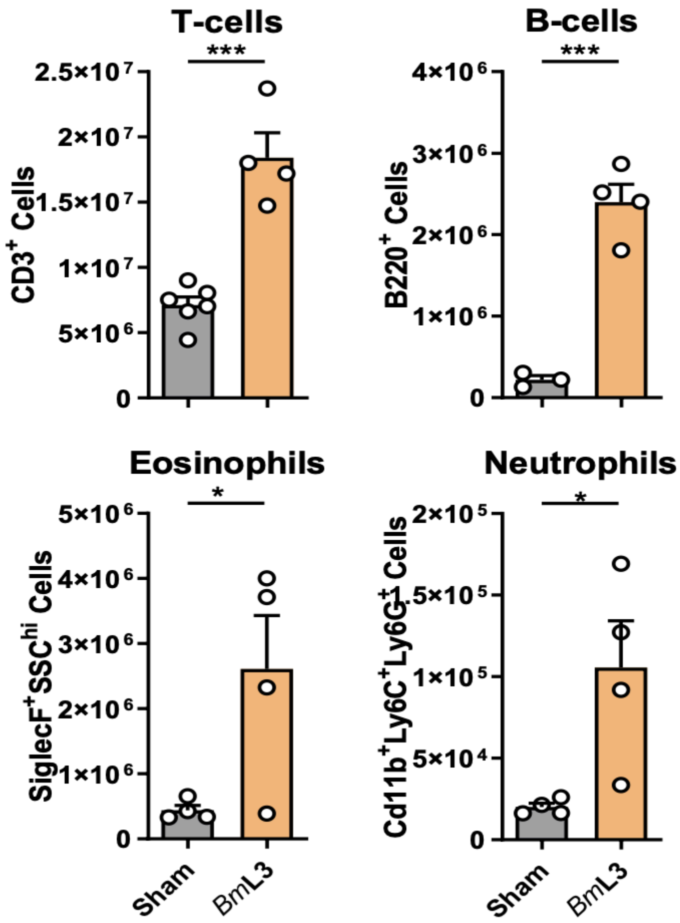

***Supplemental Figure 5** BmL3 infection results in the local expansion of multiple leukocyte populations.*

Quantified leukocyte populations from sdLNs and adjoining lymphatic tissue proximal to initial infection sight in left hind limb, 14dpi (N=6 Sham; N=4 *Bm*L3). Bars plot the mean ±SEM from an individual experiment. Statistical significance is indicated as *=P<0.05, ***=P<0.001 derived from two-tailed student t-test between marked groups.

Supplemental Figure 6

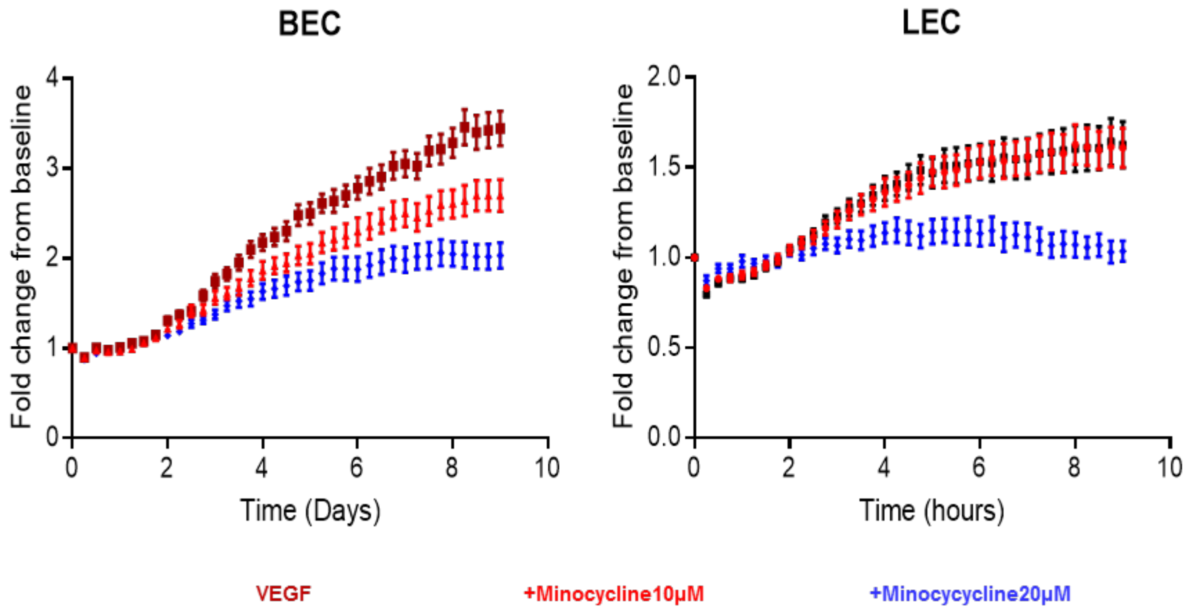

Figure S6 *Minocycline demonstrates a direct anti-lymphangiogenic mode of action* in vitro BEC and LEC proliferation growth time-courses following stimulation with 2ng/ml VEGF, in the presence of indicated concentrations of minocycline, quantified longitudinally utilising incucyte imaging technology platform. Data is plotted as fold change from day 0 BEC/LEC confluence with data points representing mean ±SEM from an individual experiment -

Supplemental Figure 7

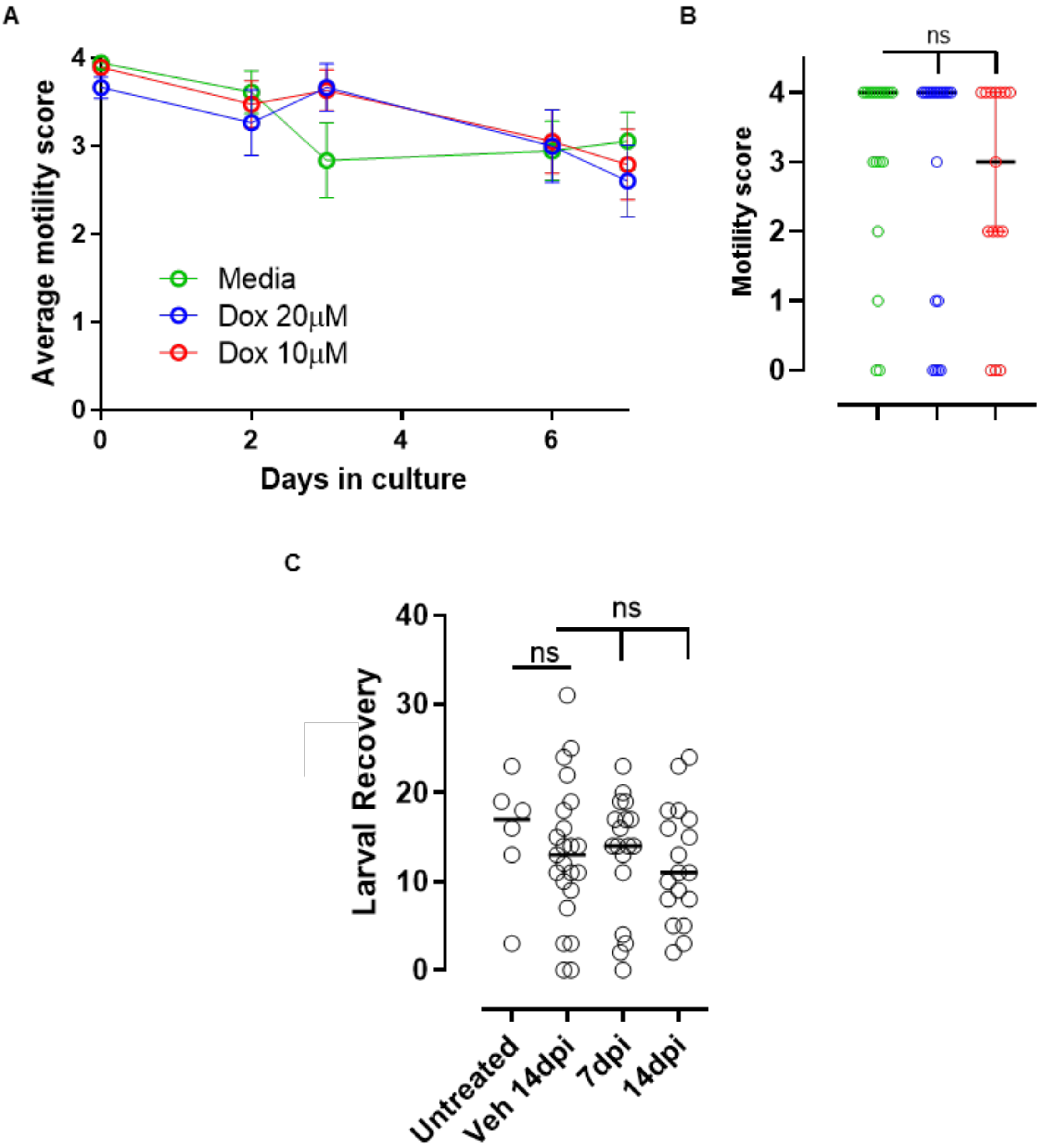

Supplemental Figure 7 *Doxycycline treatment has no significant effect on BmL3 viability in vitro or in vivo.*

A) Time course of *in vitro* cultured *Bm*L3 incubated in media alone, 10µM or 20 µM doxycycline for 7 days and individual motility scored on indicated days. The mean motility scoring of all relevant worms are displayed **B**) Endpoint (7 days post incubation) motility scoring of *in vitro* cultured *Bm*L3 (N=18 media; N=19 10µM Doxy; N=15 20 µM Doxy. **C**) Larval recovery following *Bm*L3 IP infection + : 7 or 14 day (7dpi, 14dpi) daily administration of 50mg/kg doxycycline (*Bm*L3+Doxycycline), 14 day IP administration of vehicle control (Veh 14dpi) or untreated cohorts. (N=6 Untreated; N=23 Vehicle; N= 18 7d *Bm*L3+Doxycycline; N=18 14d *Bm*L3+Doxycycline) Data is pooled from 3 (**C**) or 1 (**A-B**) individual experiments with bars representing the mean ±SEM (A,C) or median ±IQR (**B**). Significance is indicated as *=P<0.05, **=P<0.01, ***=P<0.001, ns= not significant derived from a Kruskal Wallis test with Dunn's multiple comparisons post-hoc test between indicated groups.

Supplemental Figure 8

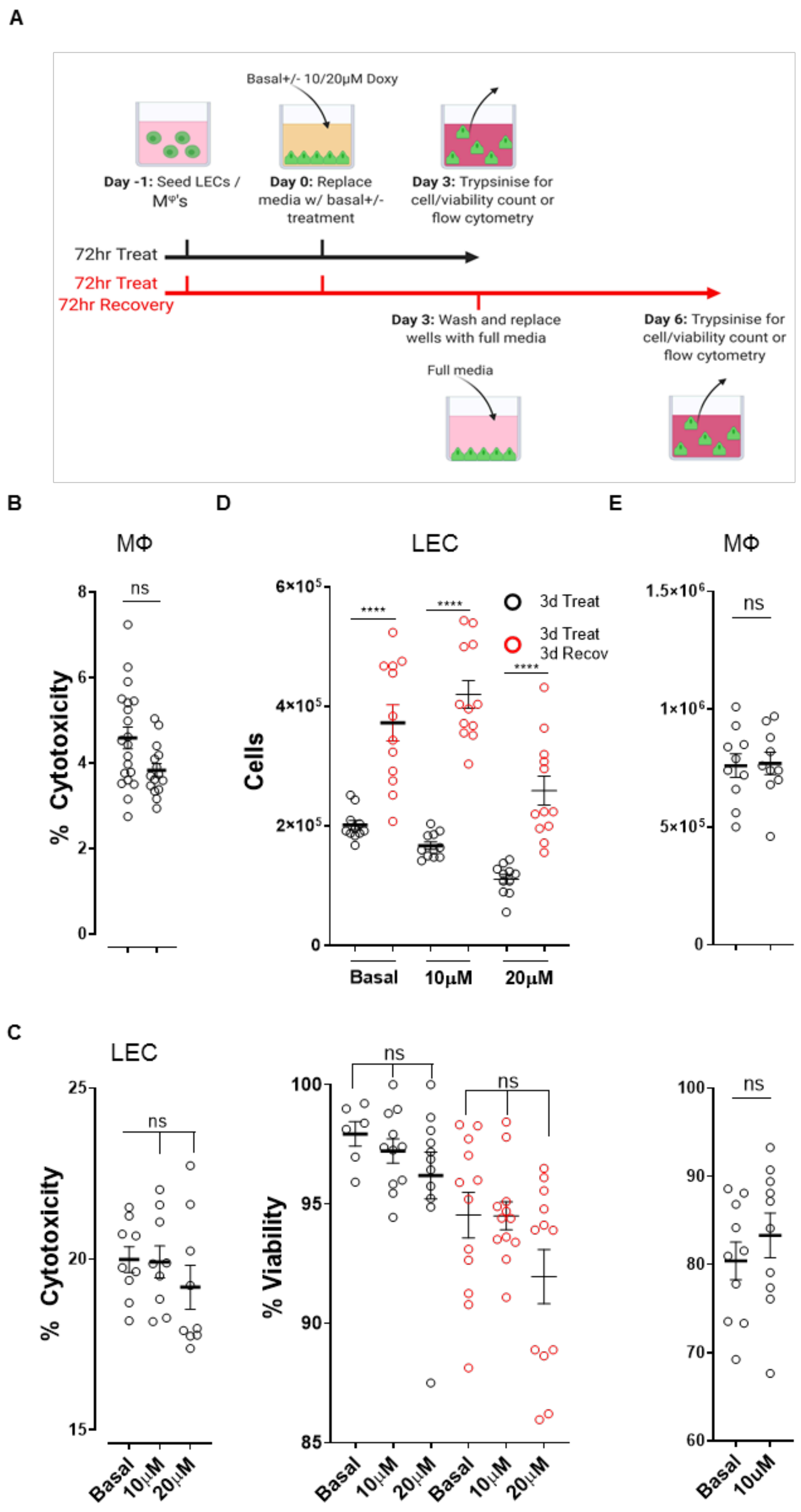

Supplemental Figure 8 *Doxycycline does not significantly affect viability of LECs or MΦs*

A) Schematic of cell treatment and recovery experiments undertaken to demonstrate viability in LECs and MΦ. Level of **B)** MΦ or **C)** LEC cell cytotoxicity by measurement of supernatant lactate dehydrogenase (LDH) concentrations and plotted as a percentage cytotoxicity of triton-x (100% cytotoxicity) treated controls **D)** LEC Cell numbers (top) and viability (bottom) folllowing 3d treatment in basal medium alone, 10μM or 20μM concentrations of doxycycline (black) or 3d treatment, followed by replacement in full media and incubation for a further 3d (red) or **E**) *MΦs* Cell numbers (top) and viability (bottom) folllowing 3d treatment.. Symbols represent single wells, bars represent mean ±SEM. Data is pooled from 2 individual experiments (**B-C**) or a single experiment (**D-E**) Significance is indicated as ****=P<0.0001, ns=not significant derived from a one-way ANOVA with Tukey's multiple comparisons post-hoc test (**B,E)** or a two tailed student t-test (**C,D)** between indicated groups.

Supplemental Figure 9

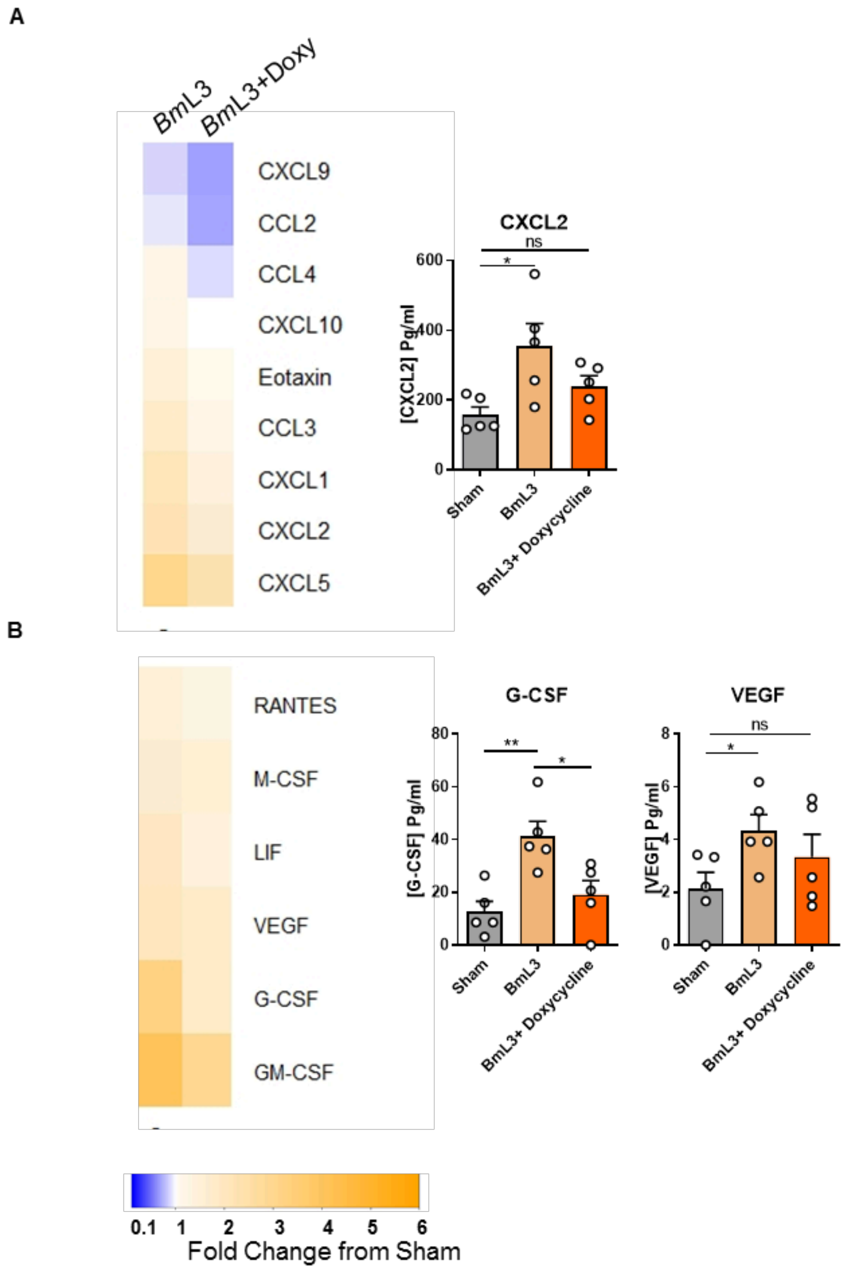

Supplemental Figure 9 *Doxycycline abrogates filarial mediated systemic increases in multiple chemokines and growth factors*

Heat maps depicting fold change increases (orange) and decreases (blue) from sham infected animals in **A)** Chemokine or **B)** Growth factor concentrations from conditioned media following *ex vivo* re-stimulation of splenocytes harvested from 14dpi sham and *Bm*L3 infected mice untreated (*Bm*L3) or treated with doxycycline. (*Bm*L3+Doxycycline) (n=5 Sham; n=5 *Bm*L3; n=5 *Bm*L3+Doxycycline). Analytes gaining statistical significance are shown on the right of the relevant heat maps. Bars represent the mean ±SEM. Data is from 1 independent experiment. Significance is indicated as *=P<0.05, **=P<0.01, ns=not significant derived from a one-way ANOVA with Tukey's multiple comparisons post-hoc test between marked groups.

Table S1

Antibody	Colour	Clone	Manufacturer
CD16/32 Fc Block	**-**	**-**	**eBioscience**
Fixable viability dye	**eFluor 450**	**-**	**eBioscience**
Ly6G	**PE**	**RB6-8C5**	**eBioscience**
Ly6C	**PerCpCy5.5**	**Hk1.4**	**eBioscience**
Rat IgG1	**APC**	**eBRG-1**	**eBioscience**
Rat IgG1	**PE-Cy7**	**eBRG-1**	**eBioscience**
Rat IgG2a	**AlexaFluor 488**	**EBR2a**	**eBioscience**
Rat IgG1	**AlexaFluor 488**	**eBRG-1**	**eBioscience**
Rat IgG1	**PE**	**eBRG-1**	**eBioscience**
Rat IgG2a	**PE**	**EBR2a**	**eBioscience**
CD4	**APC**	**GK1.5**	**eBioscience**
CCR2	**PE-Cy7**	**SA203G11**	**eBioscience**
CD206	**APC**	**MR6F3**	**Invitrogen**
CCR2	**AlexaFluor 700**	**SA203G11**	**eBioscience**
SiglecF	**APC**	**ES22-10D8**	**Miltenyi**
CD11b	**AlexaFluor 488**	**M1/70**	**eBioscience**
CD11b	**PerCpCy5.5**	**M1/70**	**eBioscience**
B220	**APC**	**RA3-6B2**	**eBioscience**
CD3	**PE**	**145-2c11**	**Biolegend**
CD3	**PerCpCy5.5**	**17A2**	**eBioscience**
F/480	**Brilliant Violet 711**	**BM8**	**Biolegend**
MHCII	**APC**	**M5/114.15.2**	**eBioscience**
Tim-4	**PE-Cy7**	**RMT4-54**	**eBioscience**
Rabbit Relm-α	**-**	**500-P214**	**Peprotech**
Rabbit Polyclonal IgG	**-**	**-**	**Peprotech**
Zenon anti-rabbit IgG	**AlexaFluor 488**	**-**	**eBioscience**
IFN-γ	**PE-Cy7**	**XMG1.2**	**eBioscience**
IL-10	**APC**	**JES5-16E3**	**eBioscience**
IL-13	**AlexaFluor 488**	**Ebio13A**	**eBioscience**
IL-4	**PE**	**145-2c11**	**eBioscience**

Table S1 *Table of flow cytometry antibodies used.*

Table with all used flow cytometry antibodies with relevant fluorophores and antibody clones

Supplementary File 1 *Representative movies from PDE imaging of sham and BmL3 infected mice, 14dpi*

Supplementary File 2 *Representative movies from Incucyte longitudinal proliferation assays demonstrating differences between VEGF and VEGF+Doxycycline stimulated BEC cells.*

Supplementary File 3 *Image J filename randomiser macro used for blinding of image analysis*

Supplementary Movie 1 *Representative movie of fluorescent BmL3 tracking experiments, demonstrating viable BmL3 inside a lymphatic channel 24h post-infection.*

Supplementary Movie 2 *Representative movie from PDE imaging of BmL3 infected mouse 14dpi, demonstrating 'splash' lymphatic pathology*

Supplementary Movie 3 *Representative movie from PDE imaging of BmL3 infected mouse 14dpi, demonstrating 'stardust' lymphatic pathology*

Supplementary Movie 4 *Representative movie from PDE imaging of BmL3 infected mouse 14dpi, demonstrating 'diffuse' lymphatic pathology*

Supplementary materials and methods

ImageJ imaging and analysis

PDE imaging was directly converted to real time imaging in ImageJ utilizing the ImageJ webcam plugin. Blinding of image stills utilised in downstream analysis was undertaken using an inhouse Filename Randomiser ImageJ macro (attached as Supplementary File 3)

ICG lymphography Image analysis

Atypical lymphatic patterns (not present in naïve mice) were quantified by tracing in ImageJ. Traced lymphatics were reported as a length measurement (arbitrary units- Pixels)- with higher lengths reporting higher quantities of aberrant lymphatic channels.

For lymphatic insufficiency measurements, a polygon selection region of interest (ROI) was used to trace round the left- and right-hind-limb in ventral viewpoint, from below the popliteal lymph node (pLN) down to above the injection site. The "mean pixel fluorescent intensity measurement" was obtained using "measure> mean grey value" tool from ImageJ (NIH, USA). An increased "mean pixel intensity", equates to higher ICG retention in the ROI. The intensities were reported as a ratio of the right (uninfected) hind lower limb: left (infected) hind lower limb, using the equation:

$$Ratio\ Value = \frac{Mean\ ROI\ Fluorescent\ Intensity\ RIGHT\ LIMB}{Mean\ ROI\ Fluorescent\ Intensity\ LEFT\ LIMB}$$

VEGFC Adenoviral infection experiments

Adenoviral vectors (ad) encoding full length VEGFC (adVEGFC) or full length GFP (adGFP) were constructed by Vector Development Lab, Texas. adVEGFC and adGFP were reconstituted in dPBS (Sigma, Dorset) and further diluted when required to create final viral dosages of $5x10^{10}$, $5x10^{9}$ or $1x10^{8}$ viral particles/injection.

C57BL/6J Prox—1^{GFP} mouse cohorts were administered bi-daily viral doses or sham control (dPBS (Sigma, Dorset) of an equal volume utilised in adenoviral injections), subcutaneously into the left hind limb as previously described. 6 days after initial injection, mice underwent PDE intravital imaging as previously described, followed by an Evan's Blue dermal retention assay. A schematic of the infection experiments can be found in figure S3A.

Cell viability and growth recovery assays

$4x10^{5}$ cells/well of LECs or $1x10^{5}$ cell/well of PMA differentiated MΦs (as described in methods) were plated in 12 well plates (Sigma, Dorset). The following day, wells were washed and media replaced with basal 80:20 (LECs) /Full RPMI 1640 media (MΦs) either alone or supplemented with 10μM or 20 μM Doxycycline (Sigma, Dorset) and incubated for 72 hours. Following 72 hour incubation, wells were either manually counted and viability assessed by

trypan blue exclusion or washed and media replaced with full EGM-MV2 media (LECs) / Full RPMI 1640 media (MΦs) respectively and incubated for a further 72 hours. Following the further 72 hour incubation, remaining wells were manually counted and viability assessed. A schematic of the assay can be found in Figure S8A

Cell cytotoxicity assays

LECS or MΦs were plated at $1x10^5$/well in 96 well plates (Sigma, Dorset), the following day cells washed, media replaced with basal 80:20 (LECs) /Full RPMI 1640 media (MΦs) either alone or supplemented with 10µM or 20 µM Doxycycline (Sigma, Dorset) and incubated for 72 hours. Following the 72 hour incubation, a proportion of wells were treated with 1% triton-x 100 and incubated for 15 minutes to serve as 100% cytotoxicity control wells. All culture supernatants were then harvested and a CyQuant LDH cytotoxicity assay (ThermoFisher) undertaken as per manufacturer's instructions. Level of cytotoxicity (LDH release) was plotted as a percentage of 100% cytotoxicity.

In vitro and *In vivo* *Bm*L3 viability assays

Freshly harvested *Bm*L3 were washed three times and plated out in 12 well plates at a concentration of 5 *Bm*L3/well in DMEM + 10% FBS +5ml Amphotericin B + 5ml Peniccilin Streptomycin (Sigma, Dorset) with no further supplementation, or supplemented with either 10 µM or 20µM Doxycyline. *Bm*L3 motility was analysed and scored microscopically on Day: 0, 2, 3, 6 and 7.

In other experiments, 100 *Bm*L3 were infected intraperitoneally into BALB/c male mice and either: left untreated, administered daily vehicle ($_{dd}H_2O$) for 14 days or treated with daily 50mg/kg Doxycycline for 7 or 14 days. Following treatment conditions described above, mice were sacrificed, and peritoneal washes undertaken to recover and enumerate *Bm*L3 numbers.
