# Supplementary figures and images for: Tetracyclines improve experimental lymphatic filariasis pathology by disrupting interleukin-4 receptor–mediated lymphangiogenesis
Source: J Clin Invest. 2021 Mar 1;131(5):e140853. doi: 10.1172/JCI140853 (PMC7919730; doi:10.1172/JCI140853)

## Slide 1
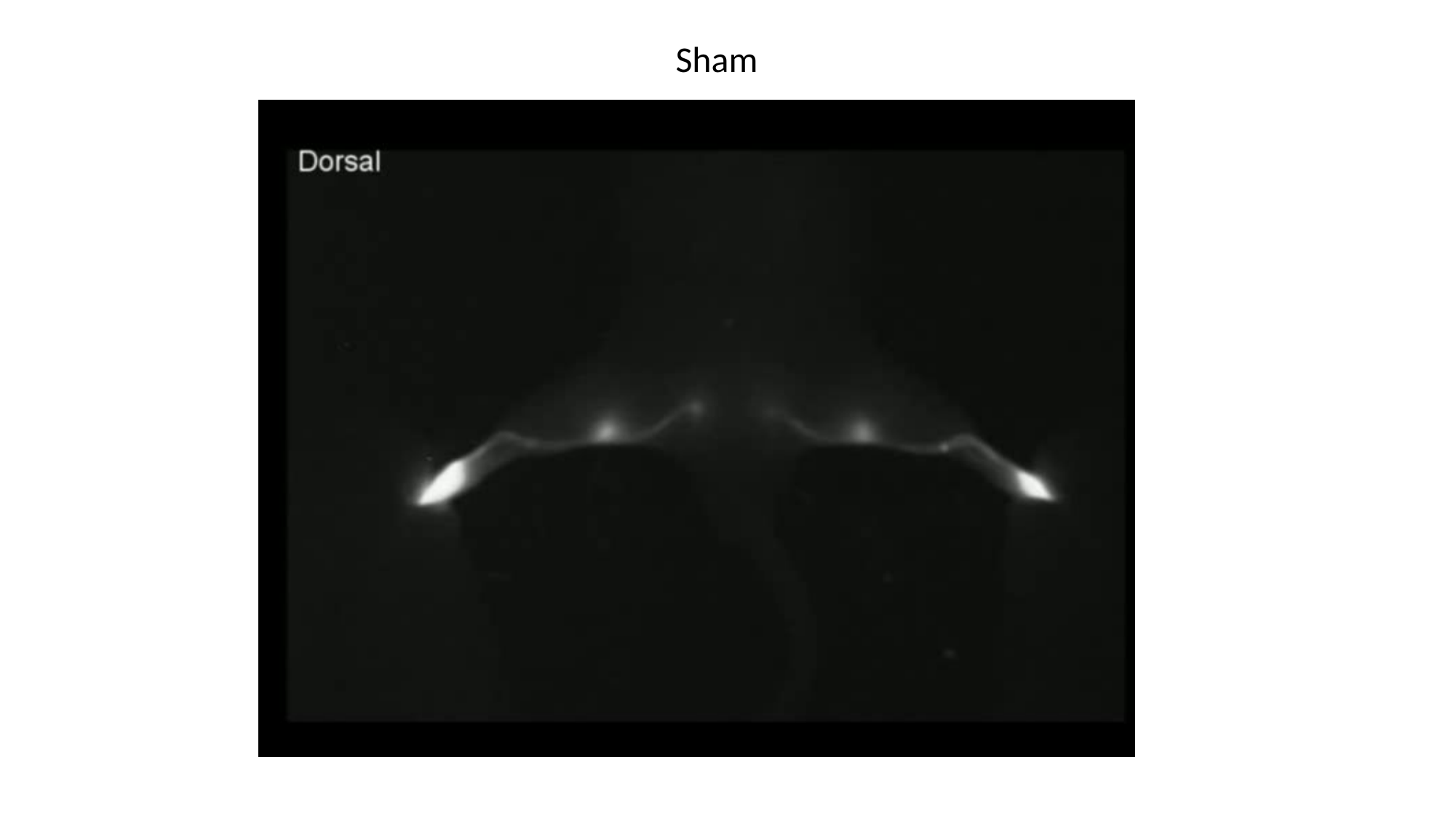

Sham

## Slide 2
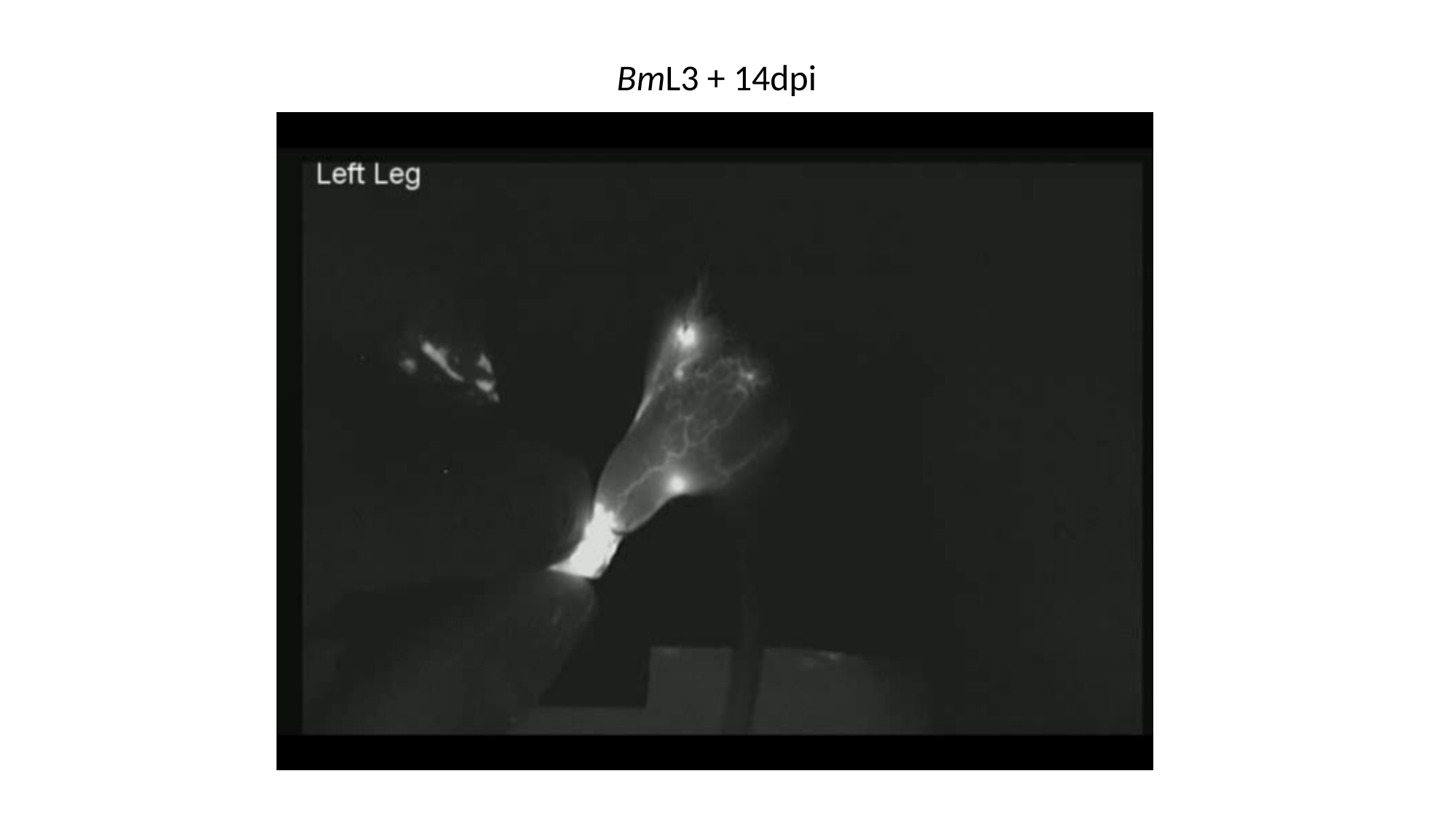

BmL3 + 14dpi

Supplement: Supplemental Data Set 1 [file jci-131-140853-s242.pptx]

## Slide 1
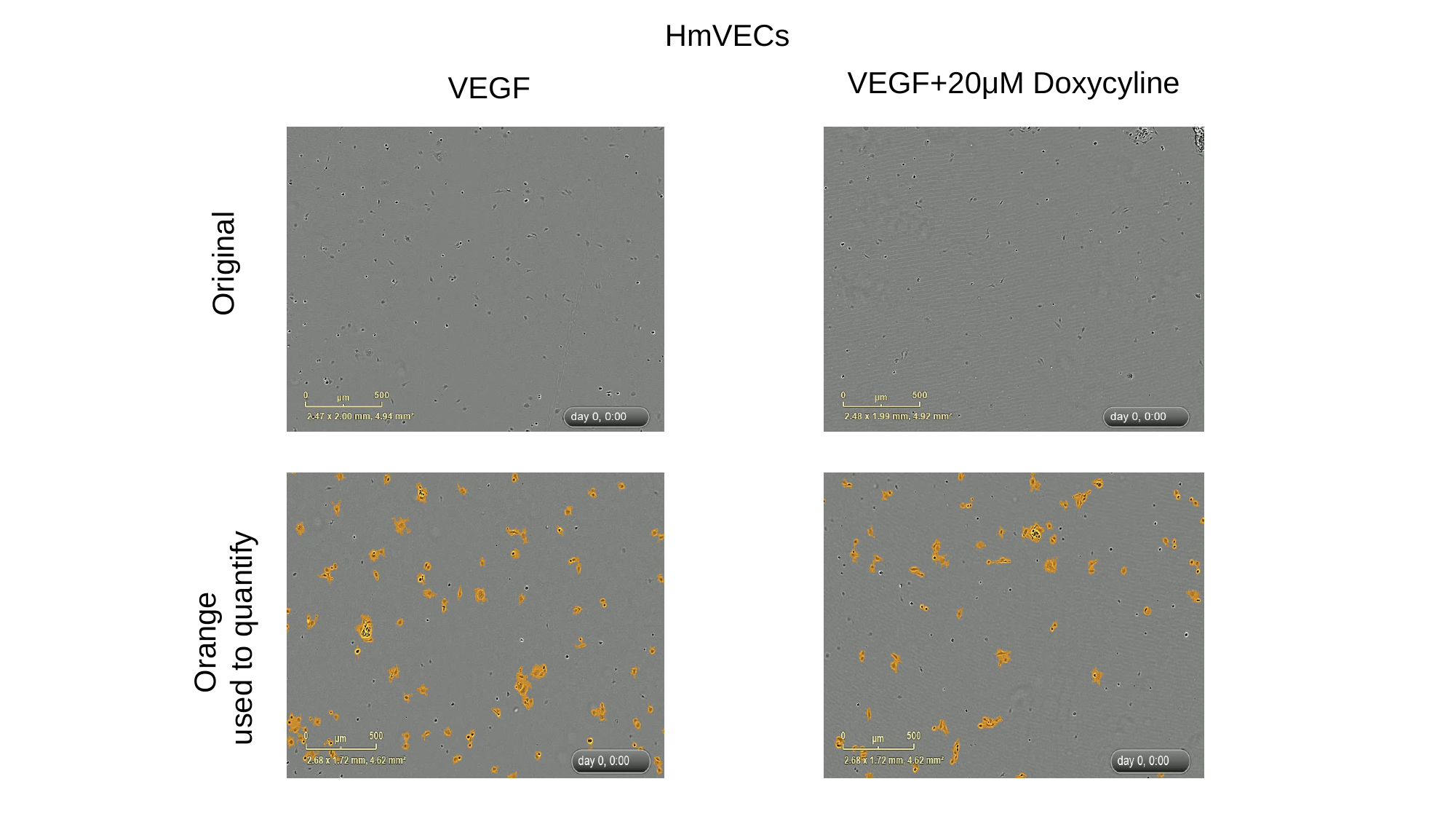

HmVECs
VEGF+20μM Doxycyline
VEGF
Original
Orange
used to quantify

Supplement: Supplemental Data Set 2 [file jci-131-140853-s243.pptx]
